# An Evaluation Framework and Comparative Analysis of the Widely Used First Programming Languages

**DOI:** 10.1371/journal.pone.0088941

**Published:** 2014-02-24

**Authors:** Muhammad Shoaib Farooq, Sher Afzal Khan, Farooq Ahmad, Saeed Islam, Adnan Abid

**Affiliations:** 1 Department of Computer Science, Abdul Wali Khan University, Mardan, Pakistan; 2 Faculty of Information Technology, University of Central Punjab, Lahore, Pakistan; 3 Department of Mathematics, Abdul Wali Khan University, Mardan, Pakistan; University of Cape Town, South Africa

## Abstract

Computer programming is the core of computer science curriculum. Several programming languages have been used to teach the first course in computer programming, and such languages are referred to as first programming language (FPL). The pool of programming languages has been evolving with the development of new languages, and from this pool different languages have been used as FPL at different times. Though the selection of an appropriate FPL is very important, yet it has been a controversial issue in the presence of many choices. Many efforts have been made for designing a good FPL, however, there is no ample way to evaluate and compare the existing languages so as to find the most suitable FPL. In this article, we have proposed a framework to evaluate the existing imperative, and object oriented languages for their suitability as an appropriate FPL. Furthermore, based on the proposed framework we have devised a customizable scoring function to compute a quantitative suitability score for a language, which reflects its conformance to the proposed framework. Lastly, we have also evaluated the conformance of the widely used FPLs to the proposed framework, and have also computed their suitability scores.

## Introduction

Computer programming holds a central importance in the computing curricula. The selection of a programming language for an introductory course of computer programming has always been pivotal as well as contentious [1], such a language is generally referred to as First Programming Language (FPL). Purpose of the first course in computer programming is to provide conceptual knowledge to the beginners for “understanding the fundamental programming constructs” in such a way that they should be able to program a given problem [2] [3]. The literature survey reveals [78] [79] [80] [81] that many different programming languages have been used as FPL. During the 1990s, Professor Richard Reid of Michigan State University has been maintaining a list of the languages used as FPL by various different universities and institutes [4]. Later on, this list has been updated till 2006 by Frances Van Scoy [79], and lately another version of this list has been compiled [78]. A summarized list of number of universities using a particular language as FPL at different times has been presented in Table 1, which has been compiled by getting data from [4] [78] [79] [80]. It clearly reflects that Pascal remained dominant FPL for a whole decade (1990s) [78], while Ada and Modula-2 remained consistent during this time. C++ gained popularity in late 90s, whereas Java and Python started to appear in the counts in late 90s. Java emerged as the most widely used FPL beyond 2006, whereas, C++ remained the runner-up throughout this time.

**Table 1:** Percentage of leading FPLs taught.

Language	YEAR				
	1994	1997	1999	2006	2011
**Ada**	15	19	18	3	1
**C**	8	11	12	7	3
**C++**	4	20	21	22	23
**C#**	0	0	0	1	1
**Fortran**	2	2	2	0	0
**Java**	0	0	3	60	56
**Modula-2**	13	11	10	0	0
**Pascal**	40	33	30	0	0
**Python**	0	0	0	4	12
**Others**	18	4	4	4	4

**Figure 1:**
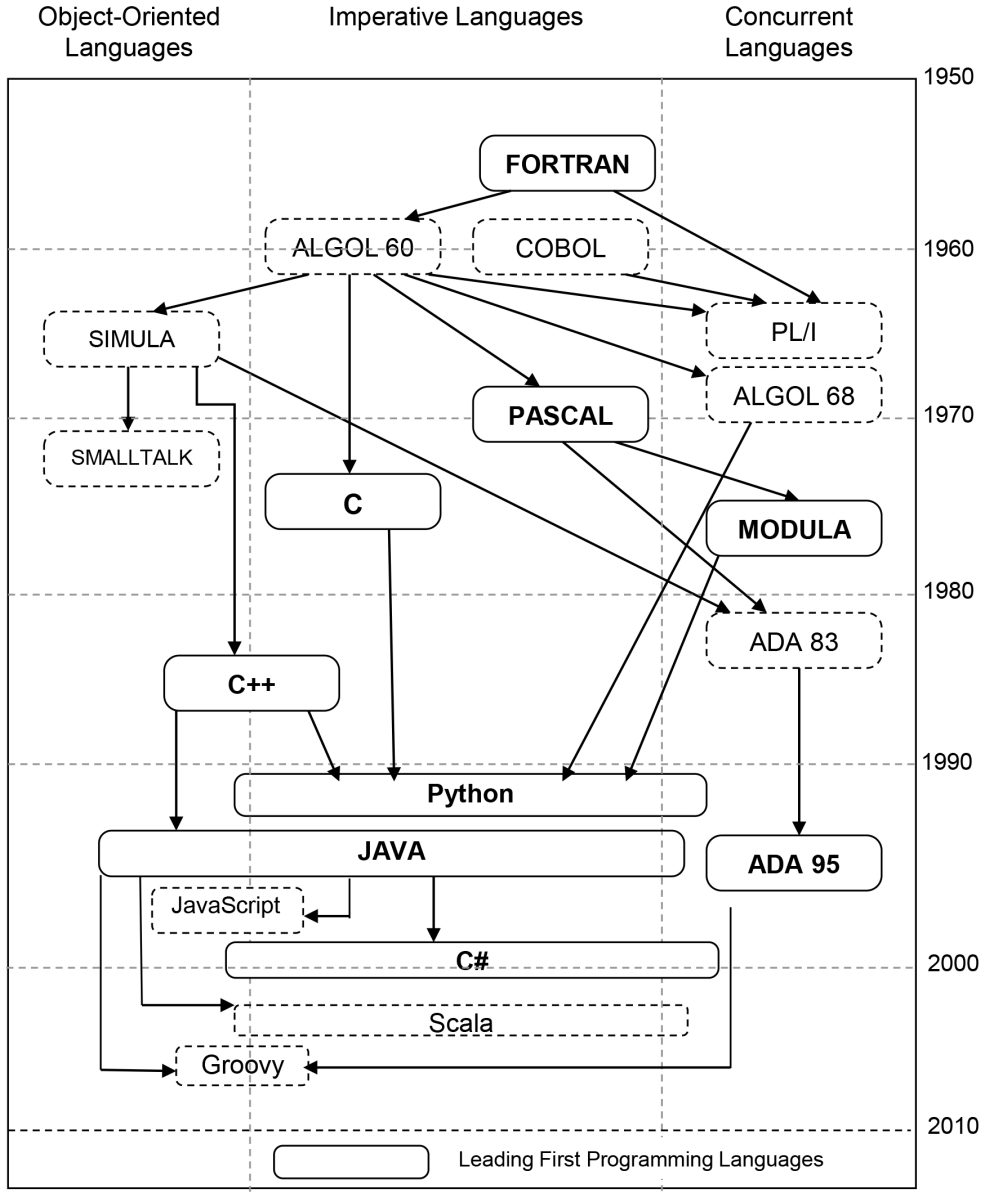
Genealogy of Programming Languages.

The genealogy of the programming languages has been presented in Figure 1. The languages in solid boxes are popular FPLs. The figure clearly shows that newer languages are influenced by some existing languages, which enforces a new language to carry some legacy features of its ancestors. Thus, the size of the new language increases, which in turn poses serious problems in terms of its suitability as an FPL. On the other hand some languages [82] have been designed purely from educational perspective, but they altogether miss out the industrial demands, and hence are not warmly welcomed by the community. This demands a comprehensive evaluation criterion for evaluating the suitability of a language as an appropriate FPL. Many people have presented different sets of requirements [5] [6] [7] [8] for an appropriate FPL. However, these approaches discuss the problem at higher abstraction levels, and to our knowledge, there is no concrete and well defined method for the evaluation of an appropriate FPL.

The major focus of this article is to figure out a possible way to evaluate the suitability of a language as an FPL. To this end, we have proposed a framework for the evaluation of an FPL which is mainly based on *technical* and *environmental* features. The novelty of this work is that while evaluating the languages we have not only relied on relevant research literature, but we have also strongly involved the general programming language rules to evaluate most of the features. We have also defined a scoring function based on the parameters in the defined framework. This scoring function is customizable and can be tuned to the user's preferences. The other contribution of this work is that we have evaluated and ranked widely used FPLs using our proposed framework. Therefore, we have considered different imperative and object oriented programming languages which have been top ranked FPLs at a certain stage. It is evident from Table 1 that Ada, C, C++, Fortran, Java, Modula-2, and Pascal have been the most frequently used FPLs, whereas, Python and C# which have recently gained popularity as FPL. The survey revealed that Scheme is also another reasonably used FPL; however, we do not consider it in our comparison as it is not an imperative language.

The rest of the paper is organized as follows: following the Introduction section, we present the related work. The proposed framework has been discussed in detail in the section *“Proposed Framework and Comparative Analysis of Commonly used FPLs”*, where we have not only presented the evaluation criterion for each feature, but we have also rated the considered FPLs over it. The scoring function along with the suitability analysis for the programming languages has been presented in the section *“Scoring Function”*. Finally, we present the conclusion and future directions of this research work.

## Related Work

Formal evaluation efforts for the assessment of programming languages are few and far between, and most evidence gathered is anecdotal in nature. Some approaches have been proposed to evaluate the quantitative suitability score for an FPL, for instance, Parker et al.[5] compiled a list of criteria for introductory programming courses at universities. However, this criterion has not been discussed with sufficient technical details of the involved measures, which can be useful for evaluation and scoring purposes.

Clarke [8] used questionnaires to evaluate a programming language. He has demonstrated that a questionnaire involving the cognitive dimensions can be a useful and valuable tool for evaluating the usability of a programming language. But, focusing just on cognitive aspects does not allow comprehensive evaluation or assessment from all aspects.

Gupta [6] discussed requirements for programming languages for beginners, which effectively is a requirement analysis for an appropriate FPL. However, there is no formal assessment mechanism devised for the evaluation of a language for its suitability as an introductory FPL. Similarly, some articles [13] [14] [17] present language independent evaluation based on intrinsic and extrinsic criterion for suitability of introductory programming language. Intrinsic criterion is related to language technical aspects such as type safety, syntax, visual *vs.* textual, compiled *vs.* interpreted. Extrinsic criterion is related external factors (student demand, industry trend), accessibility (supporting material, text books) and introductory programming course (design, thinking, algorithm social skills). However, these criterion and relevant parameters have been discussed on a surface level and need to be probed further so as to actually evaluate the languages.

McIver [7] proposed a method for comparative evaluation based on the usability of programming language. The interaction of programmer with similar language IDEs was recorded and analyzed for all types of errors made by programmer. The proposed approach by McIver evaluates languages together with similar IDEs; however, it strongly focuses on IDE and undermines the other features. Kölling [42] claims that several tools to support and improve the learning and teaching of programming have been developed, used and researched for many years, but still the problem persists.

Another dimension of research in this area is the comparative evaluation of languages that are widely being used as FPL. For instance, a comparison of Modula-2, Fortran-77, Pascal and C is presented in [10]. Phipps [11] compared C++ and Java from the viewpoint of defects, bugs and productivity rates. Similarly, Hadjerrouit examined Java's suitability as an FPL [12]. A comparison of Ada95, C, C++, and Java with their conformance to the requirements of “Steelman” has been presented in [45]. Another dimension of work, presented in [12] [15] [16] [17] by motivated faculty members, is about their dissatisfaction on a language's usability, especially, C++ and Java. This has paved way for other newer languages like Python. These articles present the evaluation of these languages based on their teaching experiences. Another recent but orthogonal dimension of work is to gather the real data about the behavior of the novice programmers [44].

The above discussion reveals that many efforts have been carried out to evaluate and compare different FPLs, yet no adequate way to assess and compare FPLs exists. This gives rise to the question of the availability of a comprehensive method to evaluate a language's strength as an appropriate FPL, which in turn, helps in comparing the suitability of different languages as FPL. In this work, we focus on defining a comprehensive evaluation criterion for the assessment of a proper FPL, with all relevant and in-depth details. The novelty of this work is that apart from defining the evaluation parameters, we have also presented the related characteristics to evaluate each parameter, and unlike existing approaches our method strongly incorporates the general programming language rules for this purpose. This effectively helps in performing comprehensive evaluation of a language, as well as may be used to compare the suitability of different languages as an appropriate FPL. We have also assigned scores to the widely used FPLs using our framework. Furthermore, we have also devised a score aggregation function so as to quantify and rank the FPLs based on the given criterion.

## Proposed Framework and Comparative Analysis of Commonly Used FPls

In this section we present our proposed framework for the suitability analysis of an FPL. Furthermore, the suitability analysis of popular FPLs, based on the parameters defined in this framework, has also been presented. To this end, we analyze each language and assign a qualitative score based on its conformance to each factor related to a parameter. Our proposed framework comprises of two main categories which include *technical* and *environmental* feature sets. The *technical* feature set covers the language theoretical aspects, whereas, the *environmental* feature set helps evaluating the external factors. These factors have been presented in Table 2. The feature sets in this framework not only help in evaluating the suitability of an FPL, but also include comprehensive guideline for designing a new FPL.

**Table 2:** Evaluation Framework.

Technical Features	High Level
	Orthogonality
	Strongly Typed
	Enforceability of Good Habits
	Security
	Feature Uniformity
	Less Effort for writing simple programs

Environmental Features	Demand in Industry
	Contemporary Features
	Easy Transition
	Readable Syntax
	Quality Coding
	User Friendly Integrated Development Environment

Firstly, we have discussed each *technical* feature separately, which are then followed by the discussion on *environmental* features, individually. We discuss each feature using the following ingredients: *(i)* define a feature; *(ii)* discuss its suitability for the evaluation of an FPL; *(iii)* define its evaluation criterion; and *(iv)* evaluate each language in Table 1 to reflect its conformance to each relevant measurable sub features for that feature.

Each feature is further comprised of a few sub features, and while evaluating each language against a feature, we rate it against each defining sub-feature. To this end, we use the following four simple qualitative values: *(i)* Fully Supported*; (ii)* Mostly Supported; *(iii)* Partially Supported; *(iv)* Not Supported. We assign an entry of “Fully Supported” to a language against a sub feature if its major implementations generally meet the requirements, whereas, “Not Supported” indicates that requirements are generally not met. The intermediate entry “Partially Supported” shows that some requirements are met, but a major portion of the requirements are not met, while “Mostly Supported” specifies that the requirement is generally met, but some specific requirements are not met. Such qualitative measures have already been used in literature for the language evaluation [45].

In order to define and evaluate each feature, we have utilized the references available in the literature; statistics related to the languages; sources considered to be language's defining documents; and the implementation of these features in the widely used compilers of that language, essentially with similar semantics. The list of documents considered for this study has been presented in Appendix S1.

### Technical Features

In this section we discuss each technical feature in detail. These technical features have been evaluated by considering a language's conformance to their defining sub-features. Furthermore, these features have also been rated against the aforementioned four qualitative values.

#### High Level

A good FPL should not have constructs that concern machine internals and possess no semantic value [18]. Jobs that can easily be managed by compiler or underlying platform should not be privileged to programmer [19]. IBM defined the level of a language as the number of basic assembly language statements it would take to produce the functionality of one statement in the target language [20]. In any high level language one instruction should be equal to three or more assembly language instructions. Table 3 describes levels of popular leading FPLs in which except C all languages are high level [20]. In our evaluation process we have incorporated IBM's criteria. For that reason C is a middle level language.

**Table 3:** No. of assembly language instructions for one instruction of the considered FPLs.

Languages	No. of assembly language instructions
Ada	6.5
C	2.5
C++	6
C#	6
Fortran	4
Java	6
Modula-2	4
Pascal	4
Python	7

It is important to note that by definition it is evaluated in quantitative terms, therefore, we do not map it to the above mentioned qualitative values. However, we treat it in a different manner, as discussed in the *scoring function* section, where we compute the overall score of a language.

#### Orthogonality

Orthogonality means all language constructs follow consistent rules [6] [9] [21] [57] [58]. As an example, in an orthogonal language keywords cannot be declared as an identifier; and semantics of statement should be predictable. Therefore, an orthogonal language offers the novice programmers a smoother and simpler learning curve. Hence, in order to evaluate the orthogonality of a language we evaluate it based on the following parameters: *i)* all keywords should be reserved; *ii)* consistent rules should be applied; and *iii)* interaction of the constructs should be predictable.

**Figure 2:**
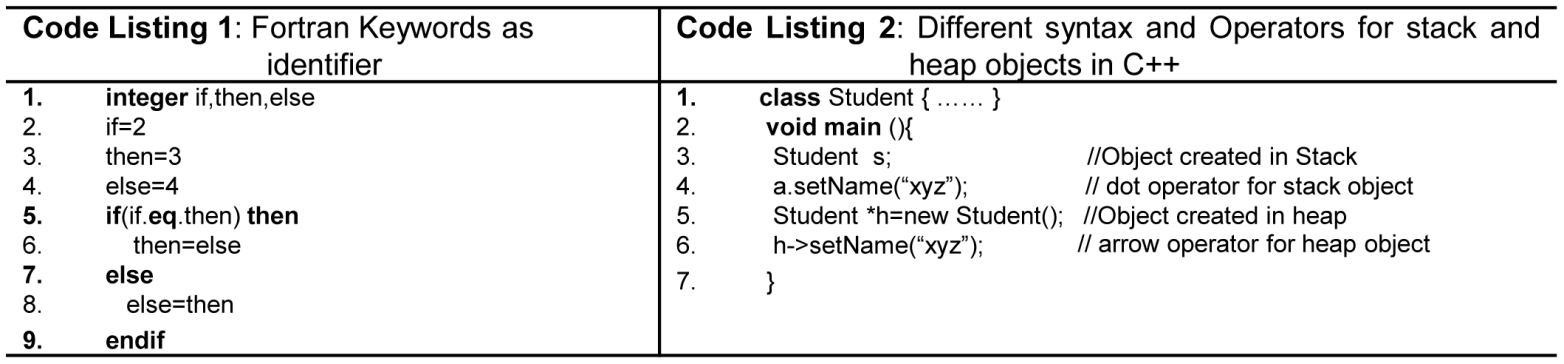
(Code Listing 1) Keywords in Fortran. (Code Listing 2) Different syntax for *stack* and *heap* memory objects.

In terms of the mainstream FPLs, all keywords are reserved in Ada, C++, C, Java, Python, Modula-2 and Pascal. However, in Fortran [21] keywords are not reserved and can be declared as an identifier. This in turn, creates serious readability problem as shown in Figure 2(Code Listing 1). C# provides two types of keywords, i.e. *reserved* and *contextual*, the *reserved* keywords can be declared as an identifier with ‘@’ prefix, while *contextual* keywords are special words for compiler in certain context and can be declared as an identifier outside the context.

**Figure 3:**
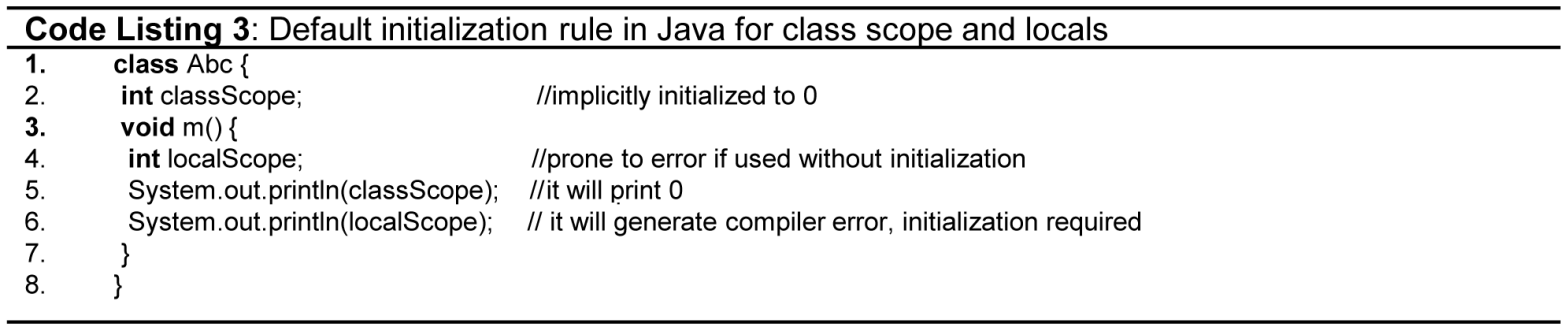
Default initialization rules in Java.

Consistent rules means that the features of a language are independent of the context of its appearance in a program. If syntactic construct is allowed for one data type, it should be allowed for all the data types available in the language, e.g. in C an *array* cannot be returned from function, but it is possible to return an array when it is placed in a structure [36] [58]. Parameter passing rule in Java is orthogonal, primitives are passed by *value*, whereas objects are passed by *reference*. All Python and Java objects are created in heap memory. In C++, objects can be created in heap as well as in stack using different syntax. The methods of objects created in stack are accessed through (.) dot operator, whereas (→) arrow operator is used to access members for the objects stored in heap, which is a violation of orthogonality as illustrated in Figure 2 (lines 4, 6 -Code Listing 2).Similarly, in Java and C#, the rule for the initialization of variables is not orthogonal. By default, all class scope primitive variables are *implicitly* initialized with its default value as illustrated in Figure 3(line 2 - Code listing 3), and objects are initialized with *null*. However, the *local* variables are not initialized by default, and should be *explicitly* initialized by the programmer as illustrated in Figure 3(lines 4,6 - Code Listing 3). However, unlike the other languages the variable initialization rule in Ada is orthogonal, as it implicitly assigns a default initial value for all types of variables and also supports *in*, *out* and *in*-*out* mode semantics for parameter passing.

Another consideration about orthogonatlity is that it enforces a predictable interaction among the constructs of a language, which means that the compiler writer is bound to implement a certain language construct as prescribed by the language documentation, and the behavior of the implementation should be documented, and needs to be consistent [45]. The defining documents for the languages C and C++ include a very large number of undefined semantics. C++ is constantly evolving and its compilers are mostly complete with respect to only a few versions, and some programs in C/C++ exhibit different behavior on different compilers [45]. Ada exhibits the same problems as some compilers have not completed their transition to newer specifications, although the validation process of Ada (including ACVC test suite) helps to ensure that its compilers implement the entire language [59]. Similarly, Fortran has also been facing the same inconsistencies as it has also been evolving over the years, and thus losing support from many compiler versions. Table 4 shows the evaluation of the considered programming languages in terms of their conformance to the property of orthogonality.

**Table 4:** Orthogonality of FPLs.

Language	All keywords are reserved	Consistent Rules	Predictable Interaction
Ada	Fully	Mostly	Partially
C	Fully	No	No
C++	Fully	No	No
C#	Mostly	Mostly	Fully
Fortran	No	No	Partially
Java	Fully	Mostly	Fully
Modula-2	Fully	No	Fully
Pascal	Fully	No	Fully
Python	Fully	Fully	Fully

#### Strongly Typed

Strongly typed means all type checking issues are resolved either at compile time, or at run time [21] [22] [57]. It ensures that no unexpected results occur at runtime due to type mismatching. Thus, it must be checked by the compiler, or by the runtime system, and no automatic conversions should be allowed. The only possible way for type conversion is explicit type casting by the programmer. Strongly typed languages are more reliable, and are easy to program and debug by novices. The concept of *strongly type* is usually implemented in two forms: *dynamic* strongly typed, and *static* strongly typed.

**Figure 4:**
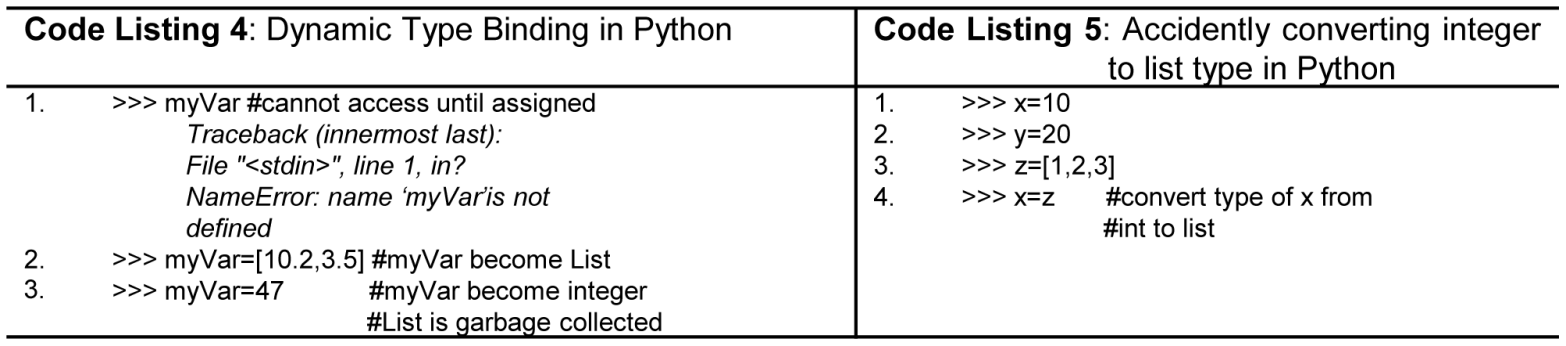
(Code Listing 4) Dynamic type binding. (Code Listing 5) Accidently converting *integer* to *list* type in Python.

In dynamic strongly typed languages the variables are implicitly declared and the type binding takes place at run time. Variables are independent of type but value has type. The variable type is determined when a value is assigned to a variable using an assignment statement, variables are *references* defined in *stack*, and *value* is an object defined in *heap*. Type of variable can be changed from one type to another type at run time as illustrated in Figure 4 (lines 2, 5 -Code Listing 4). This certainly increases programming flexibility, but at the same time, decreases early error detection. Python, JavaScript, and Ruby support dynamic strongly typed concept. In Python, incompatible types on the right side of an assignment operator are not detected as errors, rather the type of the left side is simply changed to the incorrect type. For example in Figure4 (line 1, 4 -Code Listing 5) the variables x and y store integer values, and z is storing a list. One needs the assignment statement y = x but accidently assign list z to x variable as illustrated in Figure 4(line 4 – Code Listing 5). In this case no error is detected, interpreter simply converts variable x to list type. This type of error is hardly detectable by novices, and full errors diagnostics depend on heavy unit testing, which is not possible for novice programmer.

In static strongly typed languages the variables are explicitly declared and the type binding takes place at compile time. Similarly, all errors related to type are detected at compile time. The type of a variable cannot be changed after its declaration. Fortran, Ada, C/C++, Pascal, Modula-2, Java, and C# belong to static strongly typed category. Fortran supports both *explicit* and *implicit* declarations. If a variable is not explicitly declared by programmer, then it is implicitly declared according to following convention: identifier whose name starts with I,J,K,L,M,N or their lower case versions, is implicitly declared to be an Integer; and otherwise is declared to be real.

**Figure 5:**
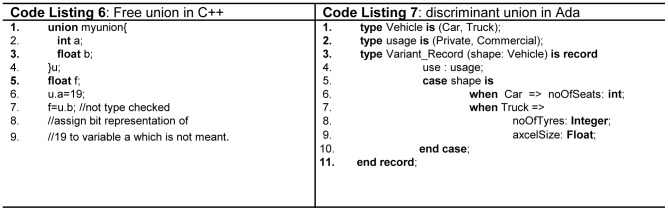
Free and discriminant *union*.

Ada allows the programmer to defer type checking for a specified type conversion using function *Unchecked_Conversion*. C/C++ are mostly static strongly typed languages, the only problem is that, the *union* construct cannot be type checked. Fortran uses e*quivalence* for *union*, and the *union* construct is not type checked. Hence, such type of union is called free *union* as shown in Figure 5 (line 7 - Code Listing 6) [21]. A special type of *union* called *discriminant union* is secure for type checking. Pascal and Ada support this type of *union* as shown in Figure 5(line 3- Code Listing 7) [21]. In Ada, Pascal, and Modula-2 *union* is type safe and more reliable. Java and C# do not support *union* due to the concerns of type safety.

**Figure 6:**
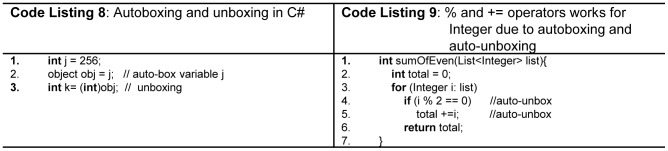
Autoboxing and unboxing.

Another type conversion issue is boxing and unboxing. Boxing is a process of converting primitive data types to object types, and vice versa is called unboxing [60] [61]. This augments a language's capability towards static type checking. C# and Java both support this concept. In C# *primitive* data types are stored onto the stack, and *object* types are stored in heap. Boxing implicitly converts stack value types to heap objects, and unboxing explicitly converts heap object to stack value as shown in Figure 6 (line 2,3 Code Listing 8). In Java boxing implicitly converts value of primitive types in corresponding object wrapper type. It will not generate some type errors. e.g. % and + =  operators are not available for Java's wrapper *Integer* type, and the compiler compiles the code without any error as shown in Figure 6 (line 4,5 Code Listing 9). Unboxing to Null object is a reliability issue as it will generate *NullPointerException* in Java. The usage of boxing concept is not recommended for arithmetic expressions; the safest way is to use this concept for the storage of primitives in collection. Java and C# boxing concept is similar to that of Python for memory allocation in stack and heap. For novices, boxing and unboxing create simplified application of heap and stack, but some types of errors are hardly detectable by the novices. In general, static strongly typed languages help the novice programmers as they help diagnosing all *type checking* errors earlier at compile time. Table 5 shows the evaluation of the considered programming language as a strongly typed language.

**Table 5:** Strongly Typed.

Language	Static Strongly Typed	Dynamic Strongly Typed
Ada	Mostly	No
C	Partially	No
C++	Mostly	No
C#	Mostly	No
Fortran	Mostly	No
Java	Mostly	No
Modula-2	Fully	No
Pascal	Fully	No
Python	No	Mostly

#### Enforceability of Good Habits

A good FPL should enforce programmers to write clean and consistent code. Good program writing style is based on clarity and readability, and these habits should be encouraged from the beginning [23] [24]. A good language should not allow:[9] [62]

1. Coercion with demotion (narrowing).
2. Expression side effects
3. Intermixing of arithmetic, logical and relational operators in Boolean expressions
4. Unconventional operator usage and overloading
5. Scope overriding

**Figure 7:**
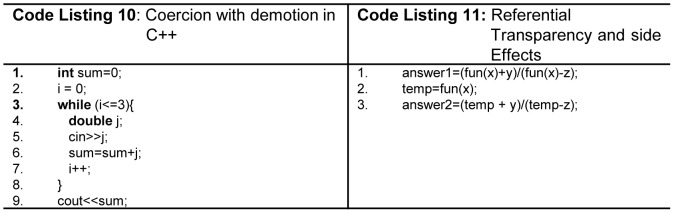
(Code Listing 10)Coercion with demotion in C++. (Code Listing 11) Referential transparency and side effects.

Coercion with demotion (narrowing conversion) results into the loss of data during the processing of *mix-mode* arithmetic expression. Fortran, Modula-2, Pascal, C++ allow promotion as well as demotion using coercion. Demotion creates data loss problem as shown in Figure 7(line 6 - Code Listing 10). Ada allows restricted form of *mix*-*mode* expressions. C#, Java, and Python discourage the concept of coercion with demotion. In terms of teaching, it is very hard for a teacher to explain *coercion* at early stages.

Arithmetic expressions are prone to get affected from functional side effects. Side effect occurs when a function changes a non-local variable or a two-way parameter [21] [62]. A good programming language produces referentially transparent programs that are more readable as shown in Figure 7(Code Listing 11). The variables *answer1* and *answer2* will be equal if function *fun* has no side effects, whereas, in case of any side effect these variables may not be equal.

**Figure 8:**
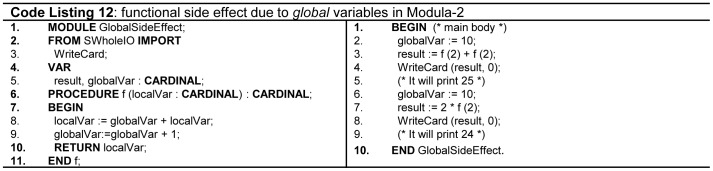
Functional side effects.

There is no concept of function side effects in mathematics, and it is also true in functional programming languages. A good programming language should not violate primitive mathematical rules. e.g. fun (2) + fun(2) is equivalent as 2 * fun(2) in mathematics. Ada supports this by using only in-mode formal parameters. *Static local* variables produce functional side effects, and Java language does not allow such type of variables. Similarly, a misuse of *global* variables violates this concept as shown in Figure 8(line 14,18 - Code Listing 12). Fortran, C, C++, Modula2, Pascal, and Python are prone to such side effects. While, due to object oriented features C++ tends to receive less use of *global* variables. Java and C# do not support *global variables* but are still prone to functional side effects. Java and C# do not support *global* variables and are prone to functional side effects.

**Figure 9:**
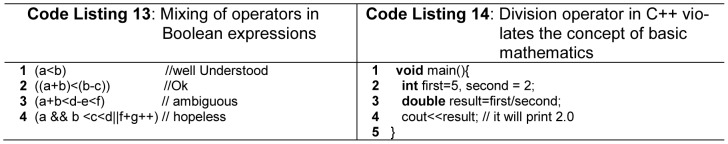
(Code Listing 13) Mixing of operators in boolean expressions. (Code Listing 14) Division operator in C++.

Mixing of arithmetic operators with logical and relational operators creates readability problem as shown in Figure 9 (lines 3,4 - Code Listing 13). It results into frustrating experiences for novices, and debugging of the code becomes tedious if arithmetic operators are used as Boolean expressions. In Mathematics, Boolean algebra *AND*, *OR* operators have same precedence, Ada supports this concept. Fortran, Modula-2, C, C++, C#, Java, and Python have different precedence for *AND* and *OR* operators. For example, in C arithmetic expressions can be written as Boolean expressions in *if* statement. In C, scalar variables (numeric or character) and constants can also be used as Boolean expressions, where ‘0’ means *false* and non-zero is considered as *true*. A good language should avoid these issues [36].

Unconventional operator usage in language design will also create readability problem. For Example, division operator (/) in most of the languages is used for integers as well as real numbers as shown in Figure 9(line 3 - Code Listing 14). In mathematics (/) operator means real division. In assignment statement *double result = first/second;* both operands in division are integer type so integer division truncates fractional result. Destination variable *result* is double so integer result is coerced to double. Here implicit type conversion (coercion) will not be responsible for the data loss. Pascal, Modula-2 and Python provide separate operators for *integer* and *real* division. Pascal and Modula-2 use *div* for integers and ‘/’ for real numbers, whereas Python uses “//” for *integer* and ‘/’ for *real*. Other languages use ‘/’ as overloaded operator for both *integer* and *real* division. In short, a good FPL should not violate core mathematical rules.

**Figure 10:**
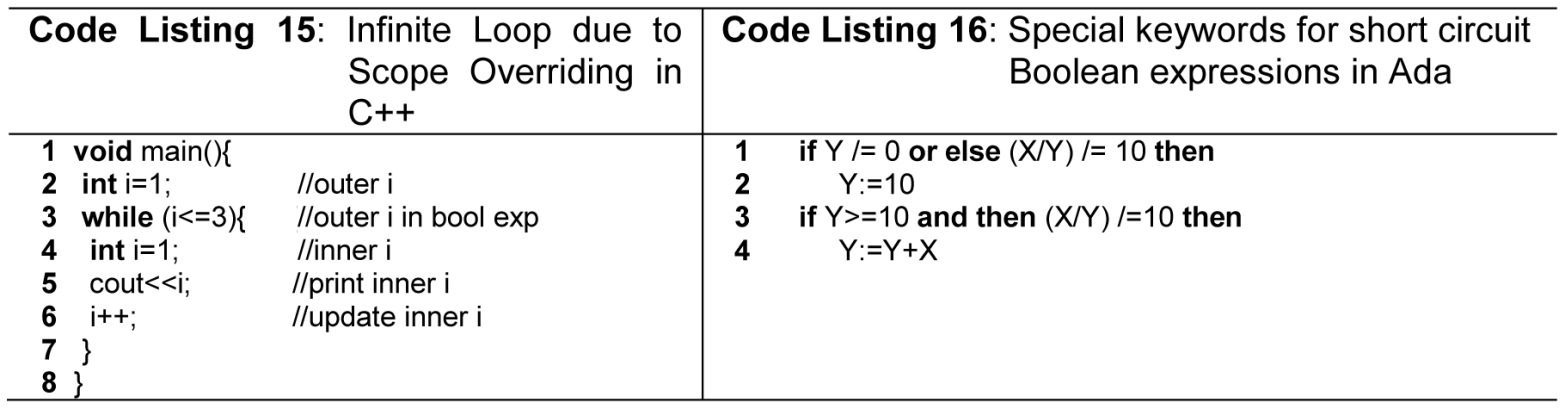
Scope overriding and short-circuit evaluation.

Unconventional operator overloading also create ambiguities. Languages which support abstract data types like Ada, C++, C#, Python, and Fortran95 allow programmer to overload operators. When used for conventional purpose it will enhance readability but unconventional use of operator overloading will create poor readability. For example use of + operator to compare two stack type objects. In order to avoid unconventional operator overloading, Java does not support this feature. Sometimes overloading of AND and OR operators overrides the default behavior of short circuiting in boolean expression, that is why C# and Python do not allow overloading of these operators. Ada defines *“and then”* and *“or else”* as short circuit form and these forms cannot be overloaded as shown in Figure 10 (line 1, 3 -Code Listing 16).

Scope overriding decreases the readability of a program. In most block scope languages, variable name should be unique with in single block, but nested blocks can declare same name variable as parent block. C++ provide scope resolution operator (::) for accessing overridden global variables. In order to avoid scope overriding problem variable names should be unique within single as well as nested scopes. For novice programmer identical names in nested blocks are too error prone and difficult to debug as shown in Figure 10 (line 2, 4 -Code Listing 15). Here Loop condition depends on outer *i* declared at line 2 but inner *i* declared at line 4 overrides outer *i*. Therefore, outer *i* cannot be accessible within the body of *while* loop, which results into an infinite loop. C# and Java does not allow scope overriding. A good programming language and coding standards should not allow scope overriding, as it is error prone, especially, for novice programmers [21] [46]. Table 6 shows the evaluation of the considered languages based on their enforceability of good habits.

**Table 6:** Enforceability of Good Habits.

Language	Coercion without demotion	No expression side effects	No scope overriding	No intermixing of operators	Restricts Unconventional operator Usage and Overloading
Ada	Fully	Mostly	No	Fully	No
C	No	Partial	No	No	No
C++	No	Mostly	No	No	No
C#	Fully	Mostly	Fully	No	No
Fortran	No	No	Fully	Fully	Fully
Java	Fully	Mostly	Fully	No	Fully
Modula-2	Fully	Partially	Fully	No	Fully
Pascal	No	Partially	Fully	Fully	Fully
Python	Fully	Mostly	Fully	No	No

#### Security

In order to evaluate a language for its strength in security we propose the following parameters: *i)* language should avoid dangling references; *ii)* there should not be any memory leakage; *(iii)* control over array index out of bound; *(iv)* pointers arithmetic; *(v)* prevent stack and heap overflows. Every programming language should support controlled aliasing. Aliasing can be brutal to the security of the program [30], and a programming language is considered to be less secure due to uncontrolled aliasing. Uncontrolled aliasing is a major threat as it may create problems like dangling referencing and memory leakage. Programs that have the keyword *new* without a matching *delete*, creates these types of problems. Best practice is to add *new* keyword and remove *delete* keyword from a language. Particularly, from an FPL's point of view inclusion of *new* and *delete* affects the pedagogical activities both in terms of teaching, and learning. Thus, the provision of automatic garbage collection should be made available in good a programming language.

**Figure 11:**
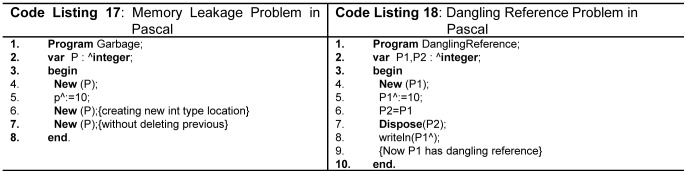
Memory leakage and dangling reference.

Dynamic memory management mechanism of Pascal and Modula-2 poses problems like memory leakage as shown in Figure 11 (line 9 - Code Listing 17), and dangling references as shown in Figure 11(line 8 - Code Listing 18).

In Java, the keyword *new* is allowed but there is no *delete* keyword. Garbage collector is responsible for deleting all non-referenced memory locations in Java, C# and Python. C++ also suffers from memory leakage and dangling reference problems since programmer has to explicitly revoke the memory using the *delete* keyword. Ada partially elevated dangling reference problem and has no solution for garbage.

**Figure 12:**
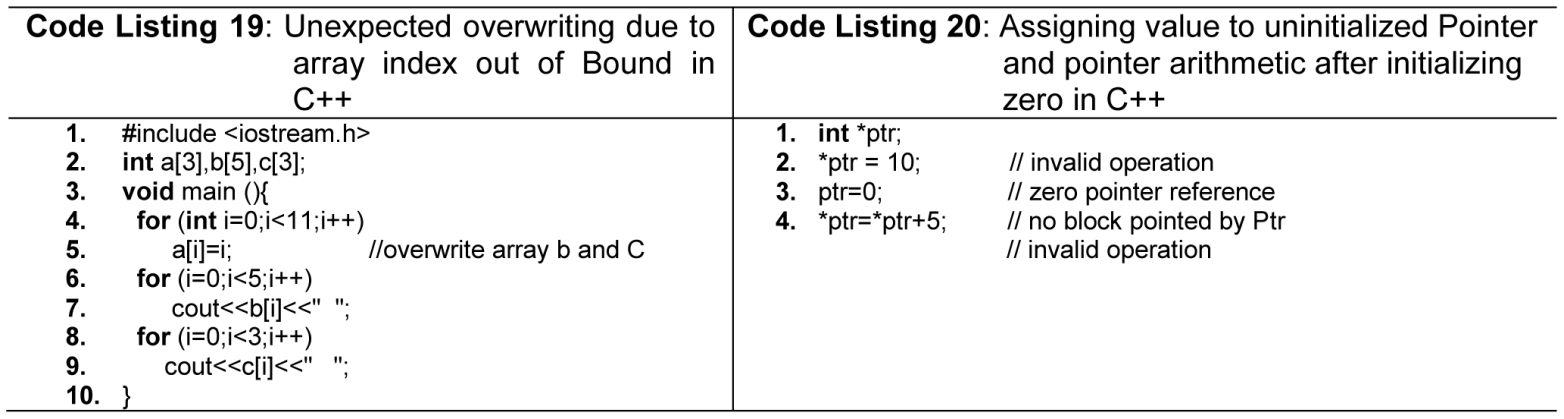
Array index-out-of- bounds, and pointer arithmetic problems.

Out of bound access in array is another security problem. C/C++ suffer from this problem which leads to read and write operations to unwanted memory locations. Novice programmers can make such mistakes as shown in Figure 12 (line 4, 5 -Code Listing 19). However, Java and C# do not pose such issues and throw array index out-of-bounds exceptions at run time, or report error at compile time. Python, Modula-2, and Ada also support this concept. Pascal addresses this issue on compile time, but cannot handle it at run time.

Another important consideration is a language's ability to detect errors related to pointer at compile time or run time. Certainly, for better diagnostics compile time detection of errors related to the pointers is much safer, and is very helpful for novice programmers as well. C/C++ have no support for detecting errors related to pointers at compile time, as well as at run time as shown in Figure 12 (line 2,3,4 -Code Listing 20). In Python all variables are considered as reference variables, which are always implicitly referenced, and direct access to the memory address is not allowed. Java does not have pointers, and only supports reference types, which can only point to objects. Java does not support pointer arithmetic on reference types. This in turn, reduces many error prone practices by novices. C# includes both references of Java and pointers of C++ using *unsafe* modifier possibly to provide backward compatibility with C and C++. Ada pointers are called *access* types and do not allow pointer arithmetic. Fortran77 does not have pointers, whereas, Modula-2 and Pascal also prone to pointer errors. Hoare [63] states about pointers, “their introduction into high-level languages has been a step backward from which we may never recover”.

Lastly, stack and heap overflows are also serious security concerns. Stack overflow mostly occurs when infinite/large recursive calls are made which consume whole memory stack. All the discussed languages suffer from this issue. Heap overflows occur when such a data array is created at runtime which requires more storage space than available memory in heap. None of the languages has been able to resolve this issues properly. All languages suffer from this issue as well. Java attempts to handle this issue by defining *StackOverflowError* in its exception hierarchy. Table 7 shows the evaluation of the considered languages based on their conformance to security.

**Table 7:** Security issues in FPLs.

Language	No Dangling Reference	Garbage Handling	Control over Array Index out of bound	Support Pointers Arithmetic	Handle Stack and Heap Overflows
Ada	Partially	Partially	Fully	No	No
C	No	No	No	Fully	No
C++	No	No	No	Fully	No
C#	Fully	Fully	Fully	No	Partially
Fortran	No	No	No	No	No
Java	Fully	Fully	Fully	No	Partially
Modula-2	No	No	Fully	Partially	No
Pascal	No	No	Partial	Partially	No
Python	Fully	Fully	Fully	No	Partially

#### Feature Uniformity

A language is considered to be feature uniform language if a proper subset of that language is not able to solve all problems that can be solved by whole set [6] [9]. Stroustrup [43] claims that in order to learn a programming language one has to learn a few fundamental constructs, techniques, and underlying models. The *minimality* of constructs certainly relaxes the learning curve. The feature uniformity in turn, can be anticipated in more than one ways, namely, feature exclusiveness and feature multiplicity [9] [21] [62].

**Figure 13:**
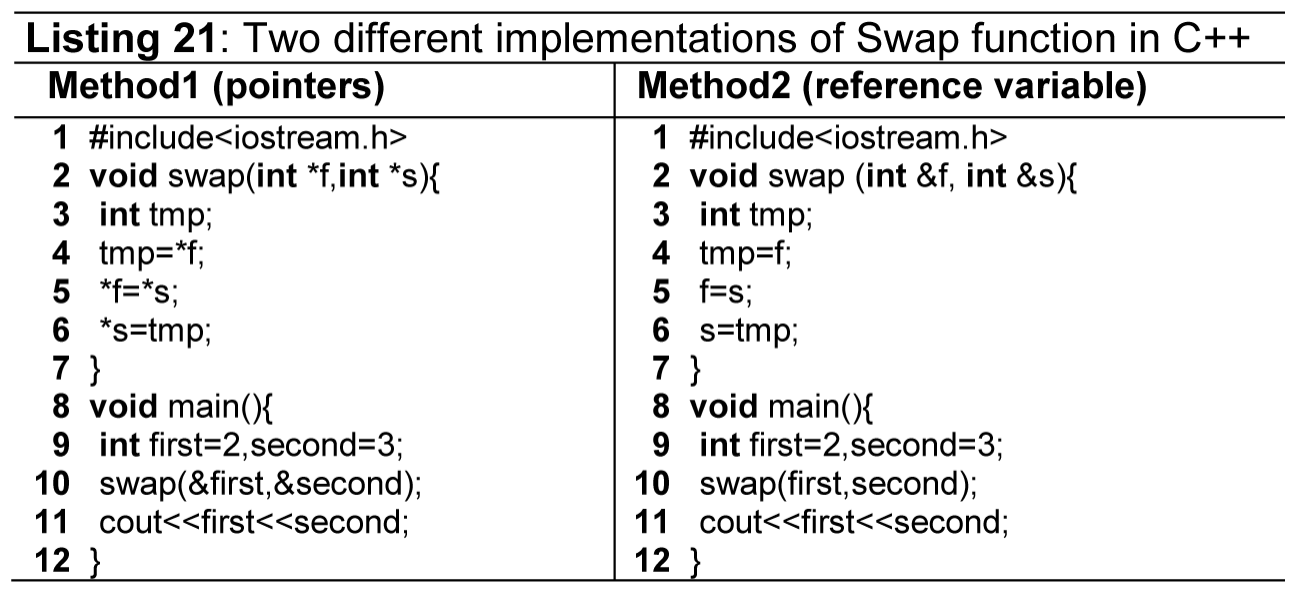
Swap function in C++.

Feature exclusiveness means a particular task can be accomplished by using exclusively different language constructs. As an example, swapping of two variables is a commonly taught programming problem in the initial programming courses. One can implement this problem in C++ using pointers, as well as using reference variables, as shown in Figure 13 (Code Listing 21). Method1 implements swap function with pointers and Method2 implements swap function through reference variables. In this case, if a novice programmer learns swapping through pointers, then in order to understand Method2, she has to learn the concept of reference variables as well, which in turn, increases the learning curve.

**Figure 14:**
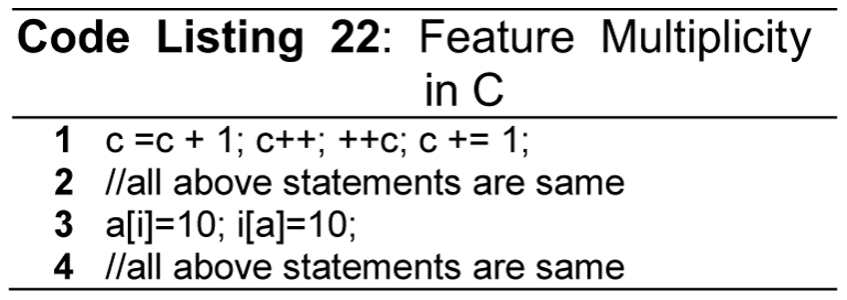
Feature multiplicity.

Feature multiplicity means more than one ways to accomplish the same task while using the same language constructs [6] [9] [21]. As an example the Figure 14 (Code Listing 22) shows the feature multiplicity for incrementing the value of a variable; and assigning a value to an index of an array.

The major problem caused by the unavailability of feature uniformity is that the size of the language increases, which in turn, results into a longer learning curve. Feature multiplicity can be observed in the control structures as they enhance language size by substituting each other. For example in C++ *while* and *for* loops are different in syntax but both have same semantics. Figure 1 shows the evolution of the programming languages, we can observe that Pascal was evolved from ALGOL60, hence, it carries the features of its predecessor language. This leads to an increase in the language complexity, in terms of the number and type of constructs. Resultantly, a language without feature uniformity usually demands the instructor to teach a subset of the language. A programmer can use this subset for writing code, but for reading other's code, comprehensive knowledge is required.

Therefore, a measure to identify whether a language holds the characteristic of feature uniformity is that its fully functional proper subset cannot be computed. Here, fully functional mean that a programmer can perform all tasks with the help of this subset. Table 8 shows the evaluation of feature uniformity for all leading FPLs.

**Table 8:** Feature Uniformity for FPLs.

Languages	Feature Exclusiveness	No Feature Multiplicity
Ada	Fully	Fully
C	No	No
C++	No	No
C#	Mostly	Mostly
Fortran	No	Fully
Java	Mostly	Mostly
Modula-2	Fully	Fully
Pascal	Fully	Fully
Python	Partially	Mostly

#### Less Effort for writing simple programs

**Figure 15:**
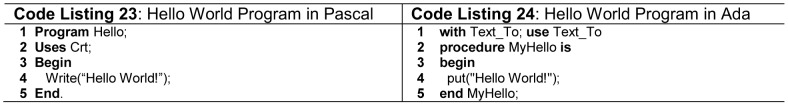
“Hello World” program in Pascal and Ada.

**Figure 16:**
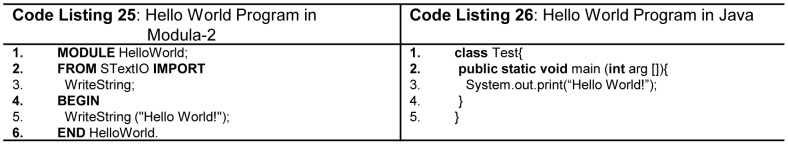
“Hello World” program in Modula-2 and Java.

**Figure 17:**
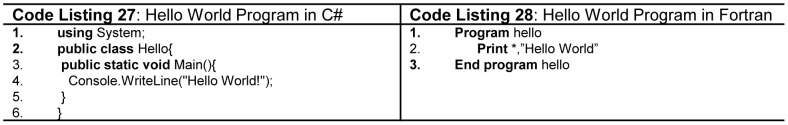
“Hello World” program in C# and Fortran.

**Figure 18:**
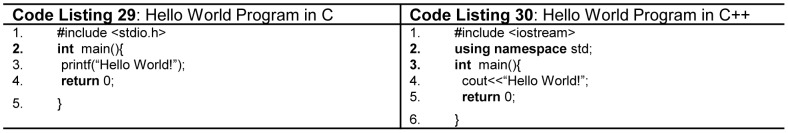
“Hello World” program in C and C++.

**Figure 19:**
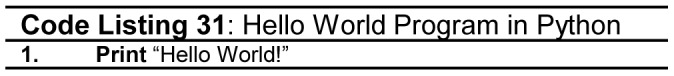
“Hello World” program in Python.

An FPL should require less programming effort to write simple programs. Furthermore, it should also support simple ways for input and output through console. In order to evaluate the effort to write simple programs we firstly consider the number of lines required to write a simple “Hello World!” program, and secondly, what is the required level of learning overhead [29]. The number of lines is fairly simple, however, we evaluate the second by identifying the number of distinct keywords used to write a simple program. For example, in Pascal writing a simple “Hello World!” program requires some learning overhead i.e. understanding of the keywords *program*, *uses, begin,* and *end* is required as shown in Figure 15 (Code Listing 23). In Ada, learning overhead (*use, with*, *procedure, begin, end)* is required for “*Hello world!*” program as shown in Figure 15 (Code Listing 24). Modula-2 also requires learning overhead (*Module, import, from keyword, STextIO library*) for *“Hello world!”* program as shown in Figure 16 (Code Listing 25). Java programmers also have to learn many basic constructs of the language for writing simple program [12] [30]. The *main* method used in Java and C# is complicated for a beginner, and is hard to explain to the novice programmers as it demands the explanation of the concepts like *class, static, public, void* etc. as shown in Figure 16 (Code Listing 26) and Figure 17(Code Listing 27). Fortran requires learning the keywords *program*, *end program* and *print as* shown in Figure 17 (Code Listing 28). C/C++ have almost same learning overhead, as C++ requires the knowledge of *namespace* as shown in Figure 18 (Code Listing 29 and 30). Python learning curve is very simple for beginners as shown in Figure 19(Code Listing 31).

The other consideration for writing simple programs is the easier use of console I/O for primitive data types. The traditional pedagogical activities in teaching an FPL involve problems that include data input from the user, and display the output to the user through console. In order to evaluate the considered languages we have incorporated a simple I/O based computer program that inputs an integer *‘a’* from the user, and displays this integer with message in the format *“value of a  = ”* followed by the value of variable *‘a’*. Table 9 shows that the code for the aforementioned problem for all considered languages. We have shown multiple ways of input for Java and C#. We have evaluated it using two considerations, firstly the learning overhead that is based on the number of lexemes; and secondly, we rate the languages higher if they possess primitive constructs for I/O than the ones which use library functions for such purpose.

**Table 9:** Console Input and Output.

Language	Console Input	Console Output
Ada	Ada.Integer_Text_IO.get (a);	Ada.Text_IO.put(“value of a = ”);
		Ada.Integer_Text_IO.put (a); Ada.Text_IO.new_line;
C	scanf(“%d”,&a);	printf(“value of a = %d\n”,a);
C++	cin>>a;	cout<<“value of a = ”<<a<<endl;
C#	**Method 1**	System.Console.WriteLine(“value of a = ”+a);
	**string** str = Console.ReadLine();	
	**int** a = Convert.ToInt32 (str);	
	**Method 2**	
	**int** a = int.Parse(Console.ReadLine());	
FORTRAN	**read** *, a	**Method 1**
		**PRINT** *, ‘value of a = ’, a
		**Method 2**
		**Write** (*,*) ‘value of a = ’, a
Java	**Method 1**	
	Scanner s = new Scanner(System.in);	System.out.println(“value of a = ”+a);
	**int** a = s.nextInt();	
	**Method 2**	
	BufferedReader keyboard;	
	**try** {	
	keyboard = new BufferedReader(new InputStreamReader(System.in));	
	**int** a = Integer.parseInt (keyboard.readLine());	
	}**catch**(IOException e){	
	System.out.println (“Error reading input!”);	
	}	
Modula-2	a: = RdInt();	WrStr(‘value of a = ’);
		WrInt(a);
		WrLn*;*
Pascal	read(a);	Writeln(‘value of a = ’,a);
Python	a = **int**(input())	Print(‘value of a = ’,a, ‘\n’)

The evaluation of console input as shown in Table 9 shows that Pascal, Modula-2, and C++ are the simplest for input. Fortran, Ada, and Python involve some additional constructs. C and C# involve even more constructs and concepts, lastly, Java offers most difficult way as it requires a lot of learning overhead for a novice.

Table 9 shows the output of most of the languages is much simpler as compared to the input methods. Pascal, Python, and C++ offer the simplest way to output data on console. Java, C#, and Fortran involve even more constructs for console output. C, Modula-2, and Ada require different instructions for different data types, which makes the output statement more complicated. Table 10 reflects the amount of effort needed to write simple programs in all considered FPLs. Table 10 shows the rating of the considered languages based on the number of language constructs to write a simple program, minimum number of constructs reflect low learning overhead.

**Table 10:** Effort required for writing simple program.

Language	Learning overhead not required	Easy Console Input	Easy Console Output
Ada	Partially	Mostly	Partially
C	Partially	Partially	Partially
C++	Partially	Fully	Fully
C#	No	Partially	Mostly
Fortran	Mostly	Mostly	Mostly
Java	No	No	Mostly
Modula-2	No	Fully	Partially
Pascal	Partially	Fully	Fully
Python	Fully	Mostly	Fully

### Environmental Features

In this section we discuss each environmental feature in detail. These environmental features have been evaluated by considering a language's conformance to their defining sub-features. Furthermore, these features have also been rated against the aforementioned four qualitative values.

#### Demand in Industry

The industrial strength of a language is that it should genuinely be capable of being used for programming in realistic industrial and commercial situations. A number of FPLs are popular in the educational institutes because of their significance in the software industry [2] [25].

In order to evaluate the industrial relevance of a language we consider the following features: *i)* the number of code repositories available online for a particular language; *(ii)* the number of available jobs; and *(iii)* the number of web searches made for a language. Different data sources available on the Internet have been used to evaluate the aforementioned three parameters. The data from *github.com (* https://github.com/munificent/github-language-ranking/blob/master/2013-08-01%20results.txt *)* has been incorporated to get the statistics about the number of code repositories for a language. Secondly, we can find the job trends from *jobstractor.com (* http://jobstractor.com/monthly-stats *)*, which provides the statistics about the jobs advertised requiring expertise in a certain language. Lastly, we have incorporated the data from *TIOBE index (* http://www.tiobe.com/index.php/content/paperinfo/tpci/index.html *)* that reflects the use of a language in web search. We believe that all these statistics help us in identifying the strength of a language for its usage and need in industry. Furthermore, all these indexes are kept up-to-date by their respective administrators, and hence can provide the language evaluator with the latest statistics, as well as, enable her to find the recent trends. We have also incorporated the latest statistics from all these data sources.

An important consideration is that all the indexes used in the evaluation of this parameter provide us quantitative data, therefore, we do not map this data on our proposed qualitative values, but we present the data in its actual form. However, we have given a special consideration to these values while computing the language's suitability score in the *scoring function* section. The recent statistics about code repositories, jobs, and number of web searches have been presented in Table 11.

**Table 11:** Demand in Industry.

Languages	No. of Code Repositories	No. of Jobs	% of Appearance in Web Searches
Ada	109	0	0
C	67706	120	18.16%
C++	78327	164	8.37%
C#	32170	343	6.02%
Fortran	1269	0	0
Java	157618	1164	16.52%
Modula-2	0	0	0
Pascal	0	0	0.72%
Python	95002	203	3.11%

It is important to note that by definition it is evaluated in quantitative terms, therefore, we do not map it to the above mentioned qualitative values. However, we treat it in a different manner, as explained in the *scoring function* section, while computing the overall score of a language.

#### Contemporary Features

Contemporary programming features and methodologies are always appealing for both academia and industry; therefore the FPL should include contemporary features based on software engineering principles [10]. These features include support of: object oriented programming, multi-threading, exception handling, packages, generic programming [21] [25] [31]. Although all such features are not taught in the FPL course, yet these features are taught in the subsequent programming courses, and are widely required in industry. Certainly, choosing a language as FPL which possesses these features reduces the transition cost of learning yet another programming language in the advanced programming courses.

Object-oriented paradigm is closest to the real world applications and is easy to understand. Object orientation is a popular and demanded feature due to its conformance to nature, reusability, and easy implementation [18] [27] [34]. C, Modula-2 and Pascal are the only widely used FPLs which do not support object oriented paradigm, whereas, the current versions of all other languages support this paradigm. Most of the popular object oriented languages, particularly the ones considered in this research, are multi-paradigm languages [21].

**Figure 20:**
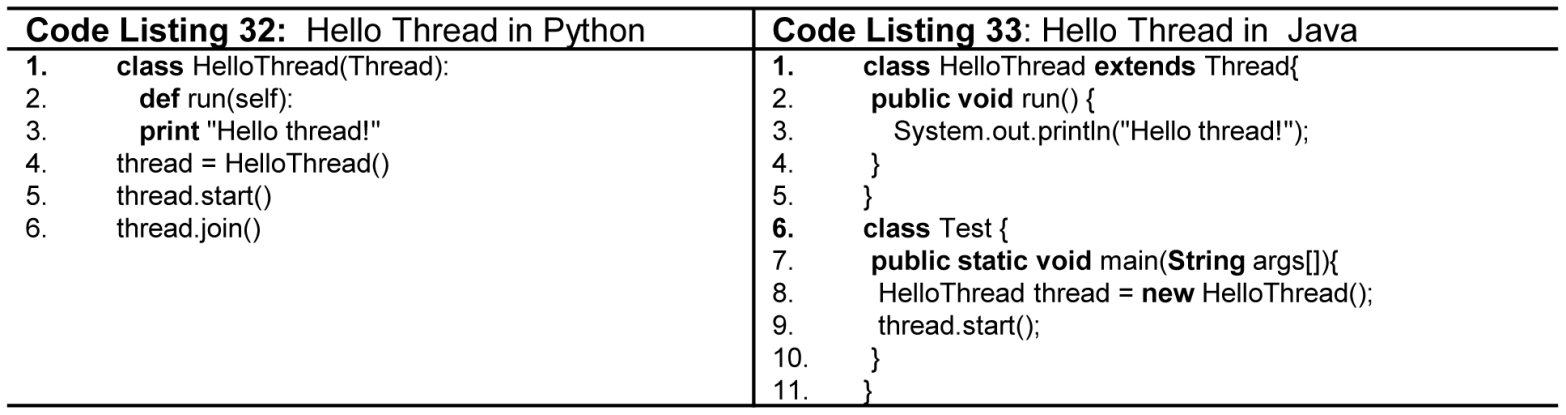
Multithreading in Python and Java.

Concurrency in Programming language [64] [65] occurs at instruction, statement, or subprogram level. Concurrency can be physical (i.e. more than one concurrent units runs simultaneously on multiple processors), or logical (i.e. more than one concurrent units run simultaneously on a single processors). In both cases, it requires synchronization, which is implemented in two ways: *competition synchronization* (mutually exclusive access to shared data), and *cooperation synchronization* (among competing tasks). All languages considered in this research work support concurrency to a certain extent. High performance Fortran specifies statements that can be executed concurrently, and also includes statements for distribution of data over memory units connected to multiple processors [66]. Ada, Java, and C# support monitors for mutually exclusive access, and semaphores for cooperation synchronization. Ada *tasks* are heavy weight tasks which communicate with each other using *rendezvous* mechanism. Java supports light weight concurrent units, any class that inherits *Thread* class or implements *Runnable* interface, and also override a method named *run,* can be executed concurrently as shown in Figure 20(line 1,2 -Code Listing 33). Here, the *competition* synchronization is implemented through *synchronized* block or method, whereas, *cooperation* synchronization is implemented through *notify*, *notifyAll*, *wait*, *join*, *sleep*, and *yield* methods. C# further improves over Java as unlike Java it allows any method to be concurrent. Furthermore, it supports three types on thread synchronization namely, *lock*, *interlock* and *monitor*. Python concurrency model is loosely based on Java as illustrated in Figure 20 (Code Listing 32). Currently, Python thread class does not support *priorities, thread groups, resume, suspend*, and *interrupt*. C, C++, Modula-2, and Pascal partially support concurrency with the help of library functions.

**Figure 21:**
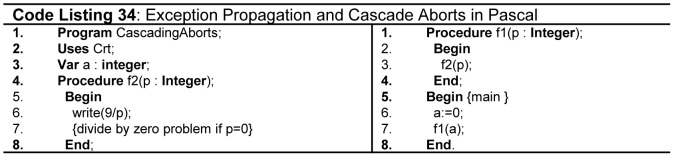
Exception propagation and cascade aborts.

Exceptions are run time unusual events, erroneous or not, detectable by hardware or software and may require special processing [21] [55]. Exception handling is a process done by code unit called exception handler. It increases the reliability as it avoids runtime failures that result into cascading aborts as mentioned in Figure 21(line 6, 11, 15 - Code Listing 34). The *write* statement in procedure *f2* will generate divide by zero *exception* for the instance where the variable *p* holds value 0. Delivering such programs to clients is highly unethical in software engineering practices. A good language should be equipped with proper exception handling mechanism.

Among the considered FPLs Fortran, C, Pascal, and Modula2 do not support exception handling. Ada supports exception handling with many problems. First, Ada exception propagation model propagates exception to outer scope from where exception is not visible and it is hard to trace the origin of error propagation. Second, its exception handling for *task* is very weak; a task without exception handling dies or raises exception. Finally, it may not always be possible to determine the object which originated the exception [67] [68] [69]. C++ is the first C-based language that includes exception handling, where exceptions are not named and are connected to handlers using actual parameter type. Formal parameter may be omitted by using ellipsis (…), in which case it catches all types of exceptions. Primitive types can be used as formal parameters in handlers, but the best practice is to define user classes for exceptions in order to enhance readability. Java supports improved form of exception handling over C++ and Ada in many ways. Firstly, only those objects that are instance or descendent of *Throwable* can be thrown as an exception. Secondly, it improves readability by introducing checked exceptions using *throws* clause in method declaration. A method without *throws* cannot throw checked exception that it does not handle. Furthermore, introducing *finally* clause for cleanup actions also enhances program readability. Lastly, JVM implicitly catches and throws variety of exceptions that can be handled by other user programs. C# handles the exceptions identically as of Java except the fact that it does not support *throws* clause. Python supports exception handling using *try, except, else, finally* keywords.

**Figure 22:**
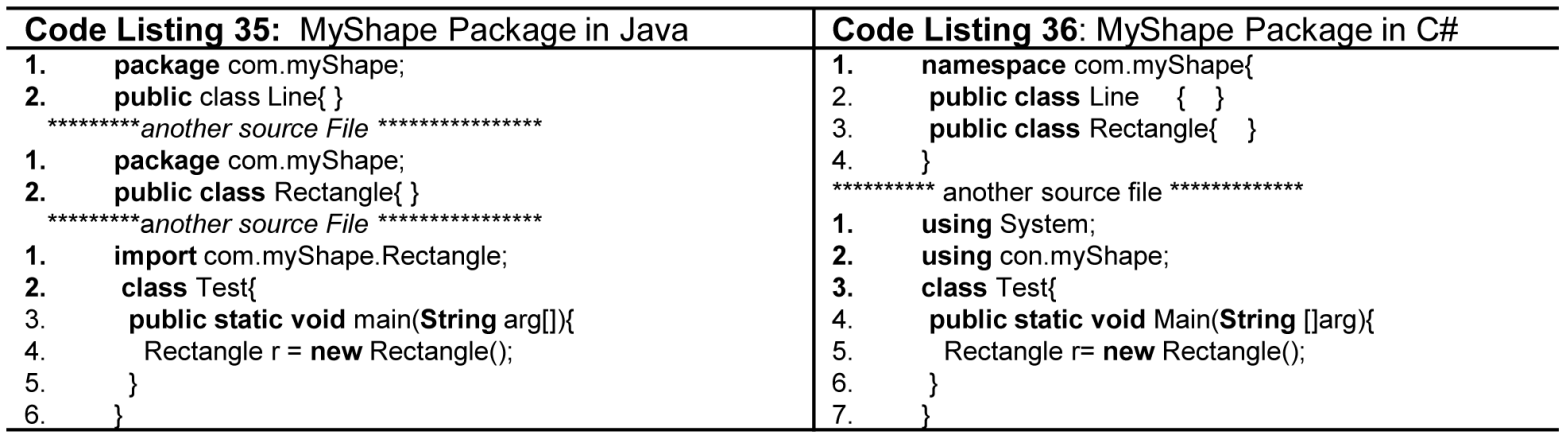
Packages in Java and C#.

Packages divide the program into manageable smaller pieces of code and provide easy ways to separately compile and assemble different pieces together to develop a large program without the inherent complexity due to its size. They also provide name encapsulation in order to define name scopes that assist in avoiding name conflicts in the APIs that expose a package to the user. Good modular design using packages supports minimum coupling and maximum cohesion [18] [28]. In Java, package is a group related public types (classes, interfaces, enumerations, and annotation), where package name dictates the source file directory structure as presented in Figure 22 (Code Listing 35). One Java package can have many sub packages. Python and Ada support packages similar to that of Java. C++ and C# provide namespace as package which unlike Java does not depend on physical layout of files in directories on disk. as mentioned in Figure 22 (Code Listing 36). The rest of languages loosely define packages: e.g. C supports packages by inclusion of header files which are stored on disk; whereas, Pascal, Modula-2 and Fortran support packages in the form of modules.

**Figure 23:**
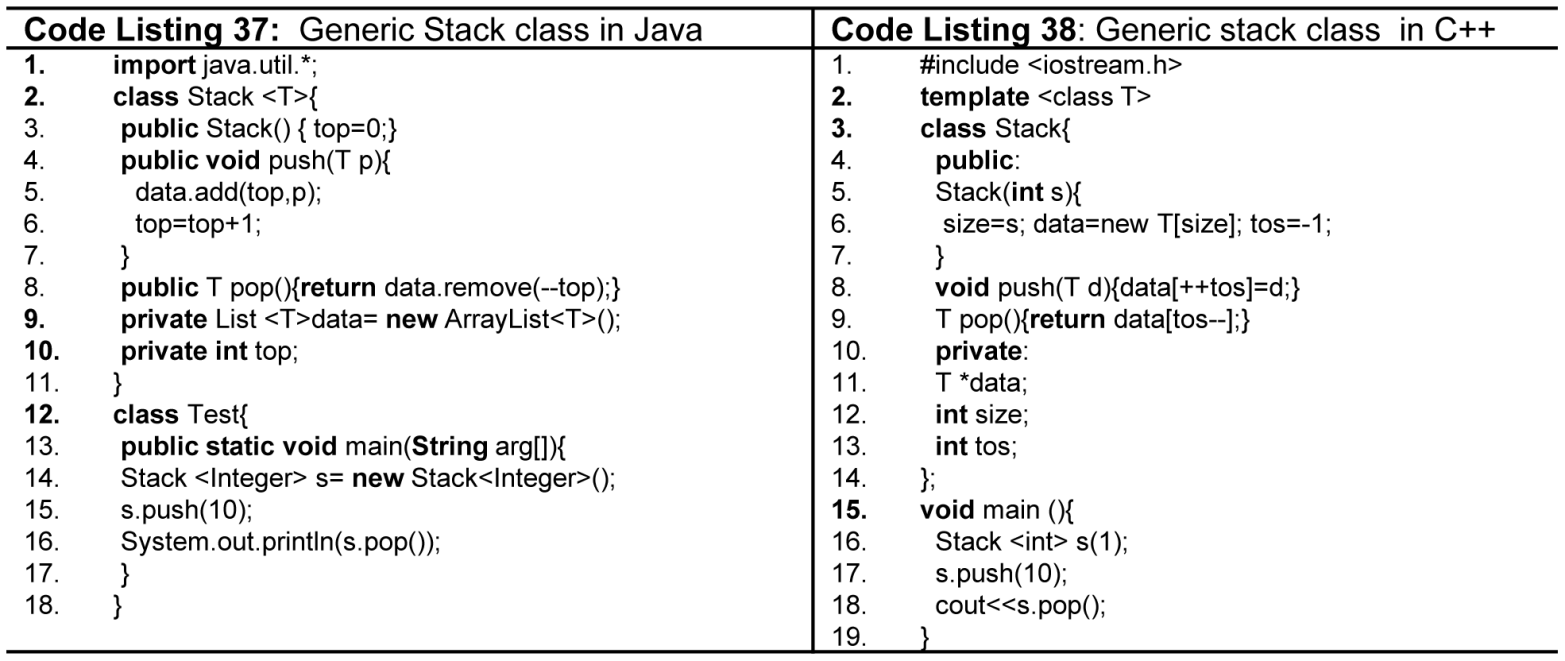
Generics in Java and C++.

Generics are subprograms or abstract data types that take parameters of different types in different activations, and are also referred to as parameterized polymorphism [21]. The use of generics offers several advantages e.g. it prevents code duplication; helps early diagnostics of errors as it converts runtime errors to compile time errors; and programmers no longer have to manually cast elements [53] [54] [55] [56]. In our considered languages Ada, C++, Java and C# support generics, while Python is dynamic strongly typed language which implicitly supports generics. Java implements generics (Figure 23, Code Listing 37) in the following different ways as compared to Ada and C++. Firstly, generic parameters must be classes not primitives. Secondly, only one copy of code is created without considering number of instantiations, called raw methods. Thirdly, restrictions can be applied to parameter that can be passed to generic method or abstract data type, called bounds. Finally, wildcards are also supported for any collection type.

C# support generics in the similar way as of Java except there is no wild card support. Ada and C++ as shown in Figure 23 (Code Listing 38) support both generic subprograms and abstract data types. A separate copy of subprogram is created at compile time for each different type, while the binding of actual and formal parameters is static. Fortran has incorporated the support of generics in its recent versions. Modula-2, Pascal, and C do not support generics. Table 12 provides the details of our considered programming languages in terms of the discussed contemporary features.

**Table 12:** Contemporary Features.

Languages	Support OOP	Support Multi-threading	Exception Handling	Support Packages	Generic Programming
Ada	Fully	Fully	Partially	Fully	Fully
C	No	Partially	No	Mostly	No
C++	Fully	Partially	Mostly	Fully	Fully
C#	Fully	Fully	Fully	Fully	Fully
Fortran	Fully	Partially	No	Mostly	Mostly
Java	Fully	Fully	Fully	Fully	Fully
Modula-2	No	Partially	No	Mostly	No
Pascal	No	Partially	No	Mostly	No
Python	Fully	Mostly	Fully	Fully	Fully

#### Easy Transition

A good FPL should allow the transition to learn any new programming language in a smooth fashion. Concepts learned with the FPL should be easily transferable to another language [32]. As an example, if one learns C++ as FPL, then the transition to Java is very smooth, since both language share many similar constructs and furthermore, these languages have almost comparable syntax. In order to evaluate our considered languages for this feature, we use three parameters. Firstly, paradigm shift is considered, i.e. shifting from imperative to Object Oriented paradigm incurs one unit of cost, whereas, vice versa does not have any cost, as Object Oriented languages are imperative. Similarly, shifting from non-concurrent to concurrent language incurs one unit cost. Secondly, a transition bears one unit of cost if the source language is statically typed, and destination language is dynamically typed, and vice versa. Lastly, the evolution of languages presented in Figure 1 is considered, which shows the influences that one language has on the other languages.

As we are computing the transition cost and certainly the language with overall minimum cost should be ranked higher. In order to map these values to our proposed qualitative measures we define a simple criterion, which assigns a category to a language based on the overall transition cost of a language to all other languages, as shown in Table 13, where ‘N’ is the number of the considered languages. The value of third parameter is equal to minimum number of hops (edges) between two languages in the evolution graph. For the total score per language we added up all values in the column see Table 14, where we present all three costs in the following format: *paradigm shift/static-dynamic type shift/hop count.*

**Table 13:** Criteria for transition cost (‘N’ is total considered languages).

	Fully	Mostly	Partially	No
	Total Cost < = 2N	2N < Total Cost < = 2.5N	2.5N < Total Cost < = 3N	3N < Total Cost
N = 9	Total Cost < = 18	18 < Total Cost < = 22.5	22.5 < Total Cost < = 27	27 < Total Cost

**Table 14:** Easy Transition (each comparison cell shows the costs paradigm shift/static-dynamic type shift/hop count).

	Ada	C	C++	C#	Fortran	Java	Modula-2	Pascal	Python	Total Cost	Rating
Ada	-	3/0/0	2/0/0	3/0/0	3/0/0	3/0/0	1/0/0	1/0/0	2/0/1	19	Mostly
C	3/1/0	-	1/1/0	1/1/0	2/1/0	2/1/0	2/1/0	2/1/0	1/1/1	23	Partially
C++	2/0/0	1/0/0	-	1/0/0	3/0/0	1/0/0	2/0/0	3/0/0	1/0/1	15	Fully
C#	3/0/0	2/0/0	1/0/0	-	4/0/0	1/0/0	2/0/0	2/0/0	2/0/1	18	Fully
Fortran	3/1/0	2/0/0	3/1/0	4/1/0	-	4/1/0	3/1/0	2/1/0	3/1/1	32	No
Java	3/0/0	2/0/0	1/0/0	1/0/0	4/0/0	-	2/0/0	3/0/0	1/0/1	18	Fully
Modula-2	1/1/0	2/0/0	2/1/0	2/1/0	3/1/0	2/1/0	-	1/1/0	1/1/1	22	Mostly
Pascal	1/1/0	2/0/0	3/1/0	3/1/0	2/1/0	3/1/0	1/1/0	-	2/1/1	25	Partially
Python	2/0/1	1/0/1	1/0/1	2/0/1	3/0/1	1/0/1	1/0/1	2/0/1	-	21	Mostly

#### Readable Syntax

The syntax of the language should be readable and consistent [26] [33]. The regular cases of errors are discovered in programs only because the programmer does not understand code written by others due to its poor readability [3]. Both beginner and experienced programmers, take advantage of good readability. In particular, for the novice programmer, it makes the learning of the language easier, helps to reduce the number of errors, and makes the code easier to maintain [34] [35].

In order to evaluate the readability of a language we use the following three parameters: *i)* Identifier's name should neither be length dependent, nor declared implicitly; *ii)* Consistent compound statement; *iii)* Meaning of constructs is not context dependent.

The first evaluation parameter for readability ensures that the names of the identifiers should not be length dependent and there should not be any implicit declaration. e.g. in Fortran 77, the length of an identifier can have 6 characters at most. It also allows implicit declaration, and identifier names starting with I,J,K,L,M,N are implicitly declared to be integer, and others are considered as real. Python infers the type of all kind of variables based on the value assigned, whereas, in C# local variables can be given an inferred type of *var* instead of an explicit type.

**Figure 24:**
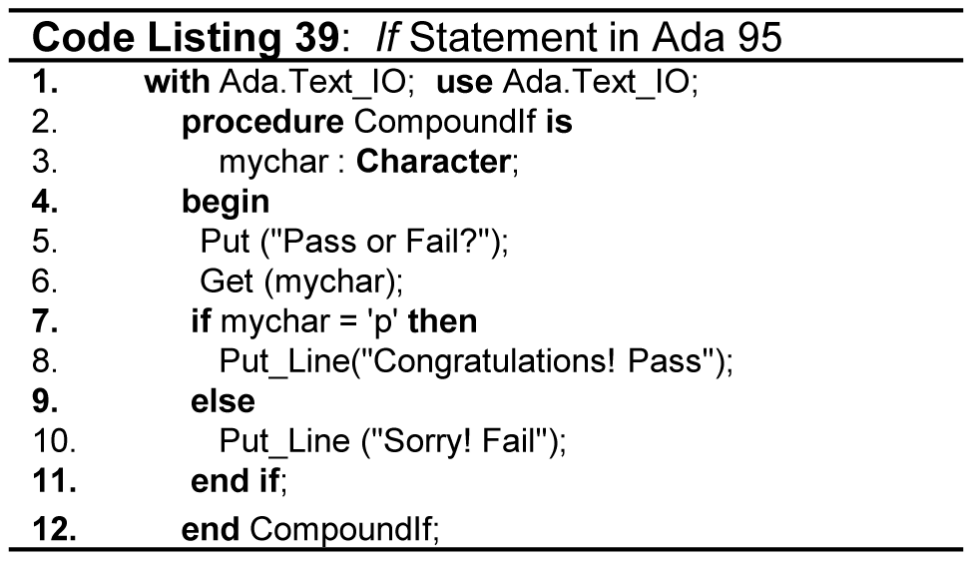
*If* statement in Ada.

There should be a clear and consistent syntax for each type of a compound statement. For instance, the usage of special keywords for signaling the *start* and *end* of each compound statement e.g. *end if* for *if* statement, *end loop* for *loop* termination, *end procedure-name* for *procedures,* and *end program* for *program* helps a great deal towards the better readability of a program. In our considered languages Fortran, Modula-2, and Ada support this feature. The code in Figure 24 (lines 4, 7, 11, 12 - Code Listing 39) shows how Ada supports this concept. The rest of the languages do not have corresponding ending keywords for compound statements, rather most of them use “{}” or “*begin end*” to represent a compound statement.

**Figure 25:**
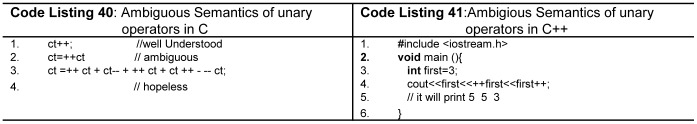
Ambiguous semantics of unary operators.

Another consideration for the evaluation of readability is that the forms and meaning of construct should not be appearance or context dependent [21]. In our considered FPLs most of the languages adhere to this requirement, however, C, C++, and Fortran do not conform to this requirement. For example, in C *static* keyword has different meaning if declared inside and outside function. Similarly, in Fortran “INTEGER :: a” is considered as declaration statement for the declaration of variable ‘a’ of type Integer, whereas, “INTEGER  =  a” is an assignment statement where the value of a is assigned to another variable named INTEGER. C# loosely conforms to this requirement as it supports context dependent keywords. Such ambiguous semantics create poor readability for novices. Unary operators are strongly discouraged due to poor readability [18] [36] as shown in Figure 25 (line 3 -Code Listing 40). Example in Figure 25 (line 4 - Code Listing 41) gives different results on different compilers. Table 15 shows the evaluation of the considered programming languages for readability.

**Table 15:** Readability of syntax in FPLs.

Languages	Identifier's name should not be length dependent and implicitly declared	Consistent compound statement	Meaning of constructs is not context dependent
Ada	Fully	Fully	Fully
C	Fully	No	No
C++	Fully	No	No
C#	Mostly	No	Mostly
Fortran	No	Fully	No
Java	Fully	No	Fully
Modula-2	Fully	Fully	Fully
Pascal	Fully	No	Fully
Python	Partially	No	Fully

#### Quality Coding Standards

The main objective of the coding standard is maintainability. Other important things that relate to the strength of the quality standards include simplicity, consistency, portability, extensibility, clarity, safety, and correctness [51] [52]. Stroustrup [41] states that production of quality code should be elevated to a central role in software development. When in doubt, the programmer should endeavor for clarity rather than efficiency [37]. The style of writing directly impacts the readability and understandability of the end product [38]. By enforcing languages to implement the coding standards we can save cost of code review and minimize the human dependency and obviously minimize the possibility of bad coding practices and explicitly improve the readability of our source code. Indentation, comments, braces, naming conventions, and parentheses are most commonly considered as quality coding standard attributes [37] [38] [39] [49]. Certainly, it is imperative to introduce the novice programmers about the coding standards from the beginning [51].

There exist coding standards like QP/C++ ™, MISRA-C++ [46] [47] which provide a guideline for writing quality code. In this work, we evaluate the quality coding standards from the perspective of an FPL, we consider the following parameters: *i)* support for comments; *ii)* avoidance of dangling else problem; *iii)* use of proper naming convention; *iv)* use of parentheses in expressions.

**Figure 26:**
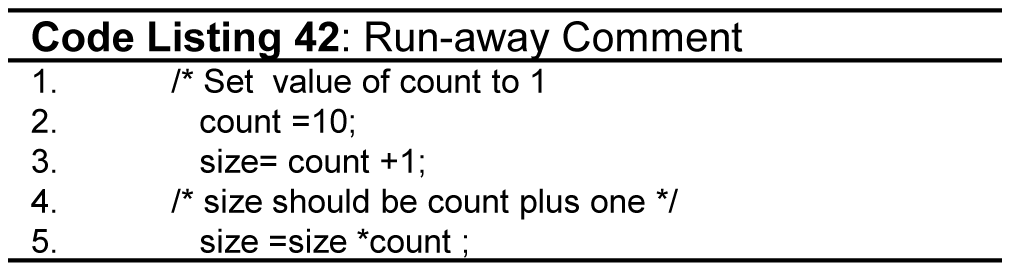
Run-away comment.

All programmers including novices are encouraged to comment the code so as to remind themselves of the tricky logic implemented in a particular part of the code. Comments are also used to provide documentation of the code. Table 16 shows differernt types of comments supported by programming languages. Generally, different types of comments are useful, but the coding standards discourage *block comments* as they are prone to errors. One serious problem in block comment is that novice programmer may accidently forget to end the comment which may swallow useful code. It will be swallowed as shown in Figure 26(code listing 42) (line 1-4).

**Table 16:** Supported comments and their types.

Language	Comment Syntax	Comment Type
Ada	— (two dashes)	End-of-Line Comment
C	/* …..*/	Block Comment
	#if 0 …..#endif	Mega Comment
C++	//	End-of-Line Comment
	/* ……*/	Block Comment
	#if 0 …..#endif	Mega Comment
Fortran	C in Position 1	Full Line Comment (now obsolete)
	! (exclamation)	End-of-Line Comment
Java	//	End-of-Line Comment
	/* ……*/	Block Comment
	/**	Documentation Comment
	*	
	*/	
Python	#	End-of-Line Comment
	“““doc String”””	Documentation Strings
Modula-2	(*…*)	Block Comment
Pascal	(*…*) Or {… }	Block Comment
C#	//	End-of-Line Comment
	/* ……*/	Block Comment
	///	Documentation Comment

**Figure 27:**
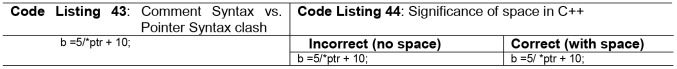
(Code Listing 43) Comment syntax vs. Pointer syntax clash. (Code Listing 44) Significance of space in C++.

Apparently, the comments may look to be a minor issue in a language; however, an unsafe comment format in a language may become a source of nasty errors particularly for novices. C++ uses/* characters for starting a block comment, while these characters are also used as multiply (*) operator, divide (/) operator, and pointer redirection. An unusual code may result into undesired erroneous programs as shown in Figure 27 (Code Listing 43), where comment syntax clashes with that of a pointer. The syntax/* denotes the start of comment and compiler will return error. Therefore, in this case the use of a *space* character is very significant. Correct code can be written with one space between/and * or use parentheses for *ptr as shown in Figure 27 (Code Listing 44). Therefore, the coding standards also discourage the usage of block comments [46].

Among different types of comments the end of Line Comment (In-Line Comments) is the most unambiguous and preferable comment [46]. Similarly, C offers *mega comments,* another type of comment, which helps in activation and deactivation of a particular part of code during execution. This type of comments is used for debugging the code. Furthermore, newer languages are equipped with documentation comments, which are useful in managing the documentation for language APIs.

In our evaluation we rate the languages higher if they facilitate the programmers with *end-of-line, documentation,* and *mega* comments, while we denounce the usage of *block* comments in a language. Although *mega* comments are useful, yet this is not considered among the mainstream types of comments. Hence, in our evaluation, we encourage the presence of *mega* comments in a language, but treat their absence unnoticed.

Based on the above discussion and supported code listings we conclude that the languages C, Modula-2, and Pascal partially support the comments. The reason is that C supports *mega* comment, but also has notorious block comment. Modula-2 and Pascal just support the block comment. Only Python supports clean comments and thus fully supports comments, while the rest of the languages have cleaner comments as well as block comments, and hence they adhere most of the comments.

**Figure 28:**
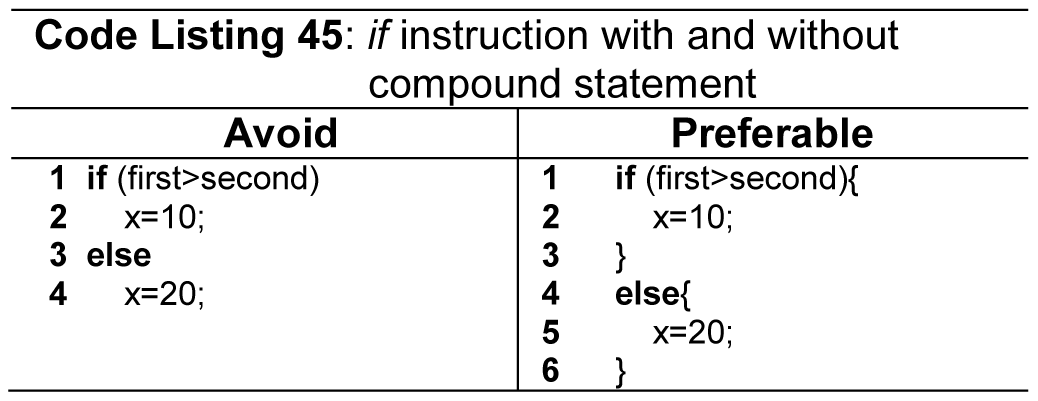
The usage of *if* instruction with and without compound statement.

**Figure 29:**
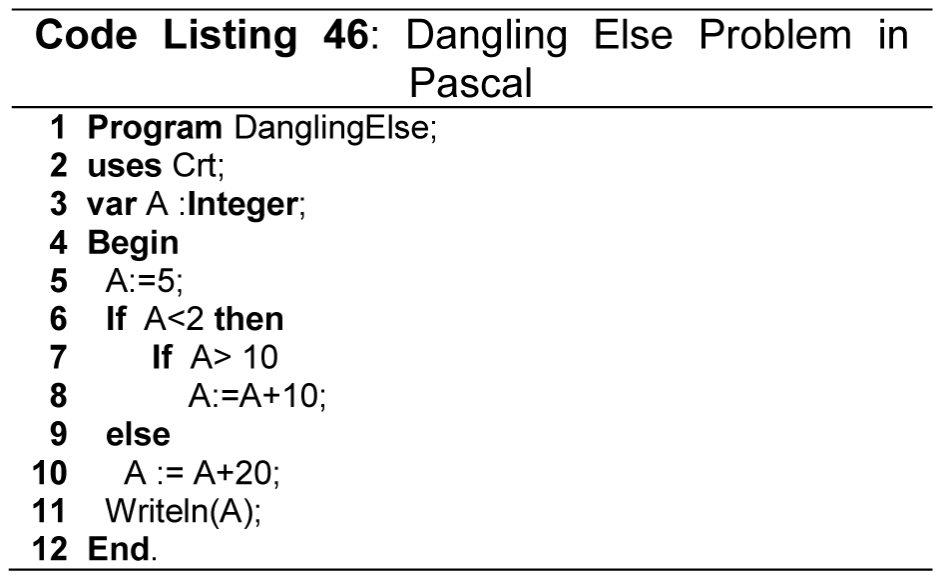
Dangling Else problem.

**Figure 30:**
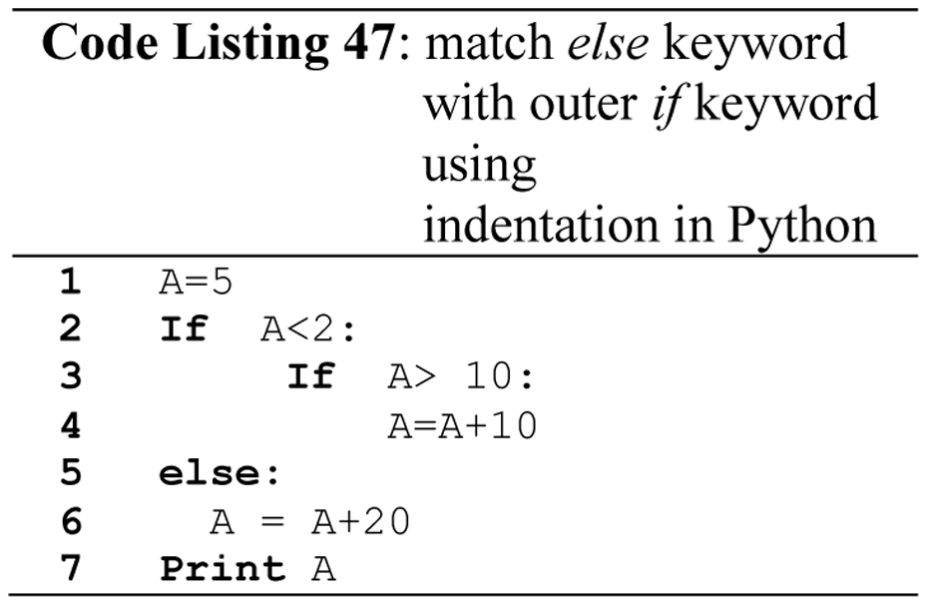
Matching of *else* keyword with outer *if* keyword using indentation in Python.

It is highly recommended in major quality coding standards to use compound statement in *if*, *else* structures and, *while* and *for* loops. For example use compound statement after *then* and *else* part as shown in Figure 28 (Code Listing 45), even if there is only a single statement in *else* and *then* part, preferable style is to use compound statement as the coding standards. MISRA/C++ [46] and QP/C++ ™ [47] also define these rules, and such rules can be verified by checkers like PC-Lint [48], and also protect code from dangling else problem as shown in Figure 28(Code Listing 45).Most of the leading FPLs suffer from dangling *else* problem as shown in Figure 29 (line 9 - Code Listing 46), where an *else* statement links with unwanted *if* statement. Python solves dangling-*else* problem by requiring indentation of *else* keyword with its matching *if* keyword as shown in Figure 30(Code Listing 47). Modula-2, Ada and Fortran solve the dangling *else* problem by using *end if* keywords. C, C++, C#, Pascal and Java suffer from dangling else problem.

**Figure 31:**
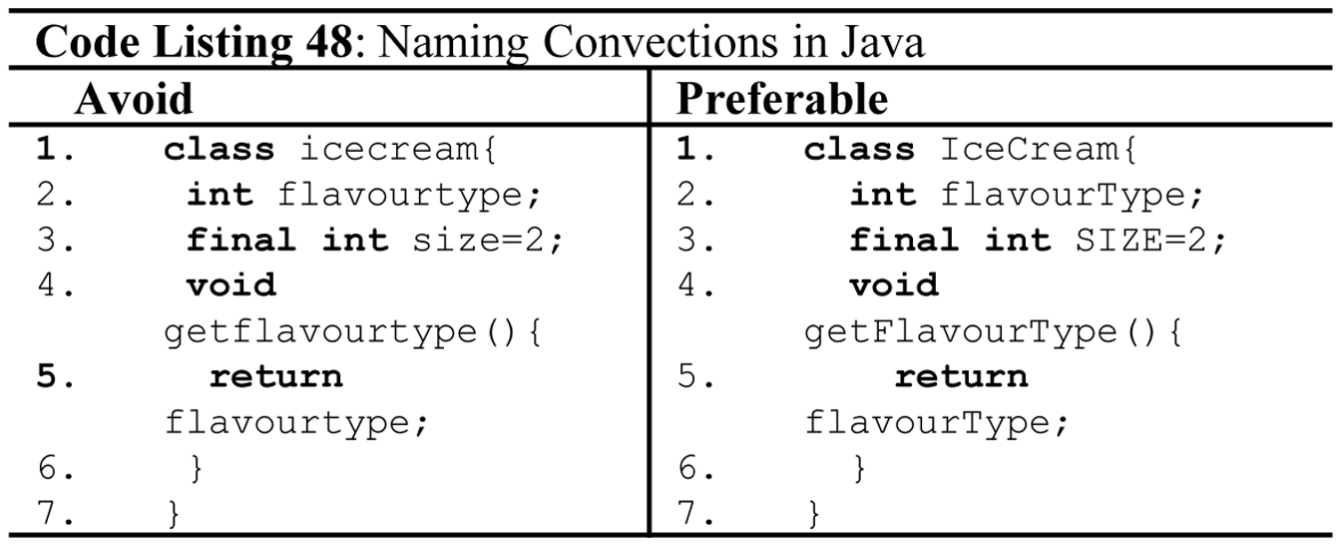
Naming conventions in Java.

The use of proper naming conventions enhances readability and comprehensibility, and it reduces the maintenance of the code [39]. Approximately, 70% of the source code of a software system consists of identifiers [70]. Knuth noted that descriptive identifiers strongly indicate the code quality and comprehensibility [71]. Java quality standard follows different naming convention for class identifiers, variables, function names and constants as shown in Figure 31 (Code Listing 48). Unfortunately, naming conventions cannot be enforced by programming languages [70], however some languages like Java and C# implicitly encourage the programmer to get used to quality naming conventions [50], as both languages involve rigorous usage of APIs which follow the coding standards.

**Figure 32:**
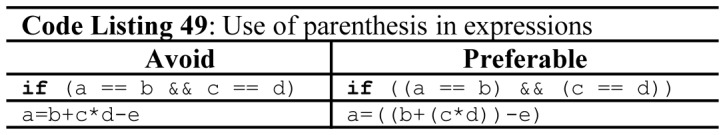
Use of parenthesis in expressions.

Use of parentheses in expressions enhances readability and code quality. Most of the coding standards enforce writing parenthesis in expressions [49] [52], e.g. if the programmer wants to write *c = (a+d)b* it will generate error. Changing it with c = a+d*b requires proper understanding of operator precedence and associatively rules. Preferable style is to use proper parentheses in expression in order to enhance code quality as shown in Figure 32 (Code Listing 49). Parentheses can easily be enforced in programming language design. It will enhance readability and help saving the novice programmer from accidental use of operators without a strong knowledge of operator associativity and precedence.

Some considerations in the coding standards cannot be enforced implicitly by a programming language but others can be enforced by changing language design as described by Table 17.

**Table 17:** Coding standards that can or cannot be implemented by programming language.

Coding Standard	Enforced by Programming Language
Indentation	Can be enforced by language design (e.g. Python)
Comments	Cannot be enforced by language design but erroneous comments (like block comment) can be removed by changing lexical design of language
Braces	Can easily be enforced by programming language through slight change in its syntax
Quality Naming Conventions like Java	Cannot be enforced by programming language
Parenthesis in expressions	Can easily be enforced by programming language through slight change in its syntax
Dangling else problem	Can easily be enforced by programming language through slight change in its syntax

None of the leading FPLs completely supports quality coding standards that can be implemented with slight modification in syntax and semantics and leaves this issue to the software engineers. Due to poor quality of code a software engineer spends many hours on code inspection, debugging, and maintenance [10]. Table 18 shows the conformance of the considered languages to the quality coding standards.

**Table 18:** Quality coding standards enforced by considered FPLs.

Language	Comments Support	Avoids Dangling Else Problem	Enforce Naming Conventions	Parenthesis in expressions
Ada	Mostly	Fully	No	No
C	Partially	No	No	No
C++	Mostly	No	No	No
C#	Mostly	No	Partially	No
Fortran	Mostly	Fully	No	No
Java	Mostly	No	Partially	No
Modula-2	Partially	Fully	No	No
Pascal	Partially	No	No	No
Python	Fully	Fully	No	No

#### User-Friendly Integrated Development Environment

In order to evaluate the user friendly integrated development environment (IDE) we consider the following parameters: *i)* structured editor; *ii)* pretty printer; *iii)* static checker; *iv)* debugger; *v)* novice programming environment. Every programming language must have a good graphically integrated development environment [7]. These environments facilitate both the novice and seasoned programmer to write, indent, and visualize the code easily. Furthermore, some researchers consider that for choosing an appropriate FPL, the programming environment alone would probably be as important as the programming language itself [7] [18] [40].

Structured editing feature in source code editor helps the programmer avoid syntactic mistakes by automatically correction and by suggesting corrections. This helps novice programmer to concentrate on problem solving rather than focusing on syntactic issues [72] [73]. All considered FPLs support this feature.

Pretty Printer handles the formatting of source code, also known as code beautifier, and involves indentation, lexeme coloring, font size adjustment, block collapse and expansion. The inclusion of such features in the text editor helps the novice programmer a great deal to improve the readability of a program [74] [75]. Except Fortran, all languages are supported by the support of pretty printers.

Static checking involves the identification of unused variables, unused functions, and violation of custom naming conventions. This helps increasing the conformance of code to the coding standards. There are several checker tools for Java (Checkstyle, FindBugs, GrammaTech Code Sonar), C/C++(CppCheck, cpplint, lint, PC-Lint), Pascal (Undertstand), Ada (AdaControl, LDRA Testbed) and Python(Pychecker, Pylint). Except Fortran all other considered FPLs facilitate static checking.

The debuggers allow a programmer to examine the state of the variables at a certain point by stopping the execution of a program. The most widely offered features of a debugger are to add watch, insert breakpoints, running program step by step, and continue execution at different locations in program. This helps the novice programmers to find errors in their programs. Furthermore, it is highly recommended that the novice programmers should learn debugging strategies [76] [77]. All considered FPLs are equipped with debuggers.

A novice visual programming environment is also imperative in terms of teaching and learning an FPL. There are several rich and user friendly novice programming environments for many considered FPLs. The list of some of the programming environments is as follows [83]: Ada (Lego Mindstorm, Ada GIDE), C/C++(BlockC, Ch), Fortan(GNOME), Java (BlueJ, CourseMaster, Greenfoot, Jeliot), and Pascal (Genie, GPCeditor, Emile, ModelIt), and Python(Alice98, Python Turtle). Some of the IDEs also support drag and drop coding (BlockC) options which help the novices to write code without syntax errors. Table 19 shows the ratings of our considered programming languages based on the provision of user friendly IDEs.

**Table 19:** Support of user friendly integrated environment.

Language	Structured Editor	Pretty Printer	Static Checker	Debugger	Novice Programming Environment
Ada	Fully	Fully	Fully	Fully	Partially
C	Fully	Fully	Fully	Fully	Mostly
C++	Fully	Fully	Fully	Fully	Mostly
C#	Fully	Fully	Fully	Fully	No
Fortran	Fully	Mostly	Mostly	Fully	Partially
Java	Fully	Fully	Fully	Fully	Fully
Modula-2	Fully	Fully	Fully	Fully	Partially
Pascal	Fully	Fully	Fully	Fully	Mostly
Python	Fully	Fully	Fully	Fully	Partially

## Scoring Function

In this section we formally define a simple scoring function for the evaluation of a programming language as an appropriate FPL. This scoring function helps in computing a quantitative score for each language, which essentially is a quantified suitability score, and reflects the strength of a language as an appropriate FPL. Previously, Parker et al. [5] presented a method to compute quantitative suitability score for an FPL. However, the criterion presented in this work has not been discussed with sufficient technical details of the involved measures, which can be useful for evaluation and scoring purposes. Our proposed scoring function considers both *technical* and *environmental* features of the proposed framework, and assigns scores to a language based on its conformance to the criterion against each parameter. We map all four qualitative measurements for each considered parameter to a quantified score using criteria given in Table 20. The mappings of *Fully* to 1, and *No* to 0 are very simple and intuitive, as 0 means no conformance, while 1 means full conformance to the criterion of a feature. In the same way, the other mappings are also supporting the criterion used for qualitative measurements as the mapping of *Mostly* to 0.66 reinforces the logic that majority of the features are being supported, and similarly, the mapping of *Partially* to 0.33 reflects that few of the requirements are justified and most of them are not supported by a language. The *technical* parameter “*High Level”,* and *environmental* parameter *“Demand in Industry”* are given a special consideration, as they are already presented in quantitative terms, so we have considered their quantitative values after bring the values to 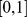 interval, by dividing all values by the maximum for each sub-feature. This in turn, makes the score values of these features compatible with the rest of features.

**Table 20:** Mapping of the qualitative measure onto the quantitative score.

Qualitative Measurement	Quantitative Score
Fully Supported (Fully)	1
Mostly Supported (Mostly)	0.66
Partially Supported (Partially)	0.33
Not Supported (No)	0

Consider a language *L* for which we need to compute the suitability score,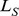, based on its characteristics. As mentioned above, the proposed framework categorizes the evaluation criterion into two main categories, technical and environmental. However, while computing the score we have grouped all parameters in one block. Based on the discussion in previous section, we map the qualitative measure to quantitative score for each parameter, using Table 20. We define the score of a language *L* against a parameter *‘i’* as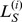.

We can also observe from the discussion in the previous section that the evaluation of certain parameters, for example, “user friendly integrated environment”, “contemporary features” etc. is based on multiple characteristics, which results into variably different values for these parameters. Therefore, while mapping the qualitative measures onto the quantitative score, the resultant score of a parameter may become unbounded, as theoretically speaking, there may be any number of sub-parameters to evaluate a particular parameter. Furthermore, the parameters with wider range of possible scores may start overwhelming the other parameters. In order to restrict the score of each parameter in a closed interval, and to avoid the aforementioned overwhelming affect, we normalize the score of such parameters by dividing the score of a parameter by maximum possible score for that parameter. As an example, the parameter “user friendly IDE” is valuated on the basis of 5 sub-parameters, and for each parameter a language can have maximum score 1, thus the score obtained for this parameter is divided by 5. This results in restricting the score value for each parameter in 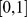 closed interval.

In reality every user may have different priorities for each parameter. Therefore, we define a weight for each parameter which a user may assign to the parameter so as to prioritize it. As an example, one may be more interested in “user friendly IDE” as compared to the “orthogonality” of a language, in which case, the scoring function allows the user to assign a higher weight to one parameter and lower to the other. By default, each parameter *‘i’* carries weight 1, i.e. 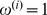. The score for parameter *‘i’* is computed by multiplying the weight 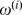 with the score of the parameter 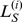, for the language L. Now, in order to compute the overall suitability score 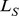 for a language *L*, we define a simple score aggregation function. This function sums the score of a language against every parameter, and the final score is computed as follows:
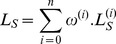



Where, *‘n’* is the total number of parameters in the language evaluation framework, which in our defined framework are 13.


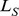
 gives us the suitability score for language *L* as an appropriate FPL. Hence, the above mentioned scoring function, and discussion in the previous section help us computing the score for all languages, and the language with maximum suitability score turns out to be the most suitable FPL.

We have further processed the suitability score by dividing the obtained score by the sum of the weights of all parameters which helps restricting the overall suitability score in the 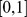 interval. This *bounded* or *normalized* score, with the default weight settings, implicitly reflects the overall percentage of conformance of a language to the proposed framework, i.e. 0.81 score reflects 81% conformance to the defined framework, similarly the difference of 0.02 should be treated as 2% less conformance. On the other hand, the benefit of using an *unbounded* score is that it reflects the differences in higher quantitative terms, but it fails to show the level of conformance to underlying proposed framework. We leave it to the user to choose any of the two score variants.
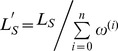



Furthermore, in order to separately highlight the strength of a language from *technical* and *environmental* perspectives we have also computed *technical* and *environmental* scores in *unbounded* (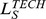,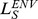), and *normalized* (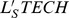,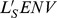) versions, as shown in Table 21. Here, *‘t’* is the number of *technical* parameter, and *‘e’* is the number of *environmental* parameters in the framework, and 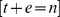.

**Table 21:** Unbounded and bounded (normalized) *technical* and *environmental* scores of languages.

	Unbounded Scores	Bounded (normalized) Scores
**Technical**	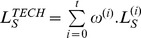	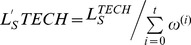
**Environmental**	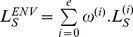	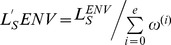

### Score Computation for the Considered Languages and Discussion

In this section, we compute the quantitative scores of the considered FPLs using the above mentioned scoring function. We obtain the scores against the parameters of each category based on the discussion in previous section. Table 22 shows the scores of all *technical* features for these languages; Table 23 shows the same for *environmental* features. Lastly, the scores of these features are combined while using the default weights in Table 24. This table, in turn, shows the suitability score for each language.

**Table 22:** Score based on technical features (sorted based on L_s_^TECH^).

Language	High Level	Orthogonality	Strongly Typed	Enforceability of Good Habits	Security	Feature Uniformity	Less Effort for writing simple programs	L_s_^TECH^	L_s_'^TECH^
**Python**	**1**	**1**	**0.33**	**0.53**	**0.67**	**0.5**	**0.89**	**4.91**	**0.70**
**Java**	**0.86**	**0.89**	**0.33**	**0.73**	**0.67**	**0.67**	**0.22**	**4.36**	**0.62**
**Pascal**	0.57	0.67	0.5	0.67	0.13	1	0.78	4.31	0.61
**Ada**	0.93	0.67	0.33	0.53	0.33	1	0.44	4.23	0.60
**Modula-2**	0.57	0.67	0.5	0.67	0.27	1	0.44	4.11	0.59
**C#**	0.86	0.78	0.33	0.53	0.67	0.33	0.33	3.83	0.55
**Fortran**	0.57	0.11	0.33	0.6	0	0.5	0.67	2.78	0.40
**C++**	0.86	0.33	0.33	0.13	0.2	0	0.78	2.63	0.38
**C**	0.36	0.33	0.17	0.07	0.2	0	0.33	1.45	0.21

**Table 23:** Score based on environmental features (sorted based on L_s_^ENV^).

Language	Demand in Industry	Contemporary Features	Easy Transition	Readable Syntax	Quality Coding	User Friendly Environment	L_s_^ENV^	L_s_'^ENV^
**Java**	**0.97**	**1**	**1**	**0.67**	**0.25**	**1**	**4.89**	**0.82**
**Ada**	**0**	**0.87**	**0.66**	**1**	**0.42**	**0.87**	**3.81**	**0.64**
**Python**	0.32	.93	0.66	0.44	0.5	0.87	3.72	0.62
**C#**	0.28	1	1	0.44	0.25	0.8	3.77	0.63
**C++**	0.37	0.80	1	0.33	0.17	0.93	3.60	0.60
**Modula-2**	0	0.2	0.66	1	0.33	0.87	3.06	0.51
**C**	0.51	0.2	0.33	0.33	0.08	0.93	2.39	0.40
**Pascal**	0.01	0.2	0.33	0.67	0.08	0.93	2.22	0.37
**Fortran**	0	0.53	0	0.33	0.42	0.73	2.02	0.34

**Table 24:** Overall score for widely used programming languages (sorted based on score with default weights).

Languages	L_s_	L_s_'
**Java**	**9.24**	**0.71**
**Python**	**8.63**	**0.66**
**Ada**	8.04	0.62
**C#**	7.60	0.58
**Modula-2**	7.17	0.55
**Pascal**	6.54	0.50
**C++**	6.23	0.48
**Fortran**	4.79	0.37
**C**	3.84	0.30

It is clear from Table 24 that Java has obtained overall highest score and thus, with default settings, it is the most suitable programming language using our defined scoring function. Python and Ada are next most suitable languages based on their obtained scores. However, one significant point is that Python is *technically* most equipped language as shown in Table 22. The reason is that it shows its strengths in many technical features i.e. it is *Orthogonal*, *High* Level, and *Secure* language, and also requires *Less Effort* in *Writing the code*. Whereas, Java ranks highest, w.r.t. the *environmental* features as shown in Tables 23, by a significant margin. This is because of the facts that Java is highly demanded in industry, supports most of the contemporary features, provides easy transitions to the other languages, and has sufficient support in the form of user friendly development environments.

Table 1 shows that C++ is still following Java as the most widely used FPL, whereas Table 24 shows that it only has 48% conformance to the defined framework with default settings. However, Table 23 shows that it has strong support from the perspective of environmental features, and Table 22 shows that it lacks strength from the *technical* feature set, as it relies on efficiency rather than reliability. However, the reason for its popularity lies in strong support for *environmental* factors, as it supports contemporary features, easy transition, and there are several use friendly IDEs for this language.

As the default weight settings do not conform to the original popularity index of the languages, so there should be a different weighting criterion. However, it is very hard to come up with a generic and correct weighting criterion. Therefore, the scoring function should be customizable and the user should be able to tune the weight of each feature based on her preferences. As an example, consider the fact that Ada holds 3^rd^ position in overall scoring, but is not being considered among highly used FPLs as of now, as shown in Table 1. The most probable reason seems to be that it fails to create any impact from the perspective of Industrial Demands, as shown in Table 23. Based on this observation a user may consider “demand in industry” and “easy transition” more important than the rest of the parameters, and assigns them weights of 3, and 2, respectively. Then, as shown in Table 25, the ranks of C#, C++, and C are elevated, whereas, Ada, Modula-2, Pascal, and Fortran are degraded with this weighting scheme, while Java and Python are not affected on the ratings list, though their degrees of conformance is affected with the new weights. This certainly shows the strength of our proposed framework and scoring function, as it re-ranks the languages based on the customized settings. Hence, every user can look for an appropriate language based on her personal preferences. However, based on the discussion in the previous section, it is clear that the user of this framework should have a reasonable understanding of the language theory to evaluate the language from *technical* perspective, and should have up-to-date information about tools, and statistics related to the language to evaluate *environmental* factors. But, the anticipated users of this framework are the personnel who are either course instructors, or curriculum designers, who in our opinion, possess sufficient background knowledge to use and customize such frameworks.

**Table 25:** Overall score for widely used programming languages (sorted based on score with customized weights).

Scores with higher weightage of “Demand in Industry” and “Easy Transition”		
**Languages**	**Unbounded L_s_**	**Bounded L_s_**'
**Java**	**12.18**	**0.76**
**Python**	**9.92**	**0.62**
**C#**	**9.15**	**0.57**
**Ada**	8.70	0.54
**C++**	**7.96**	**0.50**
**Modula-2**	7.83	0.49
**Pascal**	6.89	0.43
**C**	**5.20**	**0.32**
**Fortran**	4.80	0.30

Finally, the defined framework and scoring function are generic and flexible in two ways: firstly, any language can be evaluated on the given criterion; secondly, we have equipped the framework with many features, however, more features can seamlessly be incorporated with new advancements in the domain of programming languages.

## Conclusion and Future Directions

In this article, we have proposed a comprehensive framework for the evaluation of a programming language in terms of its suitability as an FPL. This framework mainly comprises of two main categories of features, namely *technical* and *environmental* feature sets. The *technical* features deal with the intrinsic and language theoretical aspects, whereas, the *environmental* features discuss the external factors. We have defined each feature and its corresponding sub-features, and have presented the evaluation criterion for each sub-feature. Furthermore, we have presented a scoring function to compute a quantified suitability score for a language based on our framework. In addition to this, based on the language evaluation and scoring function, we have computed the suitability scores for widely used FPLs. We have also used different parameter settings for computing the suitability scores, and give the users leverage to customize the weightage of different features, which reflects the flexibility of our proposed scoring function. This framework not only helps in the evaluation of a language as an FPL, but can also be used as a guideline for designing new FPLs.

The potential future directions of this work include the customization of a programming language so as to make it more suitable FPL w.r.t. the *technical* feature set. Similarly, our work highlights the drawbacks of the languages in terms of *environmental* feature set, and hence, new tools can be developed to increase the conformance of a language to the environmental features like *quality coding standards, user friendly environment,* and *readability*.

## Supporting Information

Appendix S1 (DOCX)
