# Supplementary material for: An Evaluation Framework and Comparative Analysis of the Widely Used First Programming Languages
Source: PLoS One. 2014 Feb 24;9(2):e88941. doi: 10.1371/journal.pone.0088941 (PMC3933420; doi:10.1371/journal.pone.0088941)
Supplement: Appendix S1 — (DOCX) [file pone.0088941.s001.docx]

**Appendix S1**

The defining documents used for each of considered FPLs are as follows:

| **Language** | **Document** |
| --- | --- |
| Ada | ISO/IEC 8652:1995Ada Reference Manual |
| C | [ISO/IEC 9899:1999 - Programming languages -- C](http://www.iso.org/iso/home/store/catalogue_ics/catalogue_detail_ics.htm?csnumber=29237)  [ISO/IEC 9899:2011 - Information technology - Programming languages-- C](http://www.iso.org/iso/iso_catalogue/catalogue_tc/catalogue_detail.htm?csnumber=57853) |
| C++ | ISO/IEC 14882:2003-Programming languages - C++  ISO/IEC 14882:2011Information technology -- Programming languages -- C++ |
| Fortran | ISO/IEC 1539-1:1997-Information technology -- Programming languages -- Fortran - Part 1: Base language |
| Java | Java™ Platform, Standard Edition 7,API Specification |
| Python | Python v3.3.2 documentation |
| Modula-2 | Niklaus Wirth, Programming in Modula-2, Fourth Edition, 1988. |
| Pascal | ISO 7185:1990, Information technology -- Programming languages – Pascal |
| C# | ISO/IEC 23270:2006-Information technology--Programming languages--C# |
